# Preoperative liquid biopsy transcriptomic panel for risk assessment of lymph node metastasis in T1 gastric cancer

**DOI:** 10.1186/s13046-025-03305-x

**Published:** 2025-02-07

**Authors:** Ping’an Ding, Jiaxiang Wu, Haotian Wu, Wenqian Ma, Tongkun Li, Peigang Yang, Honghai Guo, Yuan Tian, Jiaxuan Yang, Limian Er, Renjun Gu, Lilong Zhang, Ning Meng, Xiaolong Li, Zhenjiang Guo, Lingjiao Meng, Qun Zhao

**Affiliations:** 1 The Third Department of Surgery, the Fourth Hospital of Hebei Medical University, Shijiazhuang, Hebei 050011 China; 2 Hebei Key Laboratory of Precision Diagnosis and Comprehensive Treatment of Gastric Cancer, Shijiazhuang, 050011 China; 3 Big data analysis and mining application for precise diagnosis and treatment of gastric cancer Hebei Provincial Engineering Research Center, Shijiazhuang, 050011 China; 4 Department of Endoscopy, The Fourth Hospital of Hebei Medical University, Shijiazhuang, 050011 China; 5 School of Chinese Medicine, School of Integrated Chinese and Western Medicine, Nanjing University of Chinese Medicine, Nanjing, Jiangsu 210023 China; 6 Department of Gastroenterology and Hepatology, Jinling Hospital, Medical School of Nanjing University, Nanjing, Jiangsu 210002 China; 7 Department of General Surgery, Renmin Hospital of Wuhan University, Wuhan, Hubei 430065 China; 8 Department of General Surgery, Shijiazhuang People’s Hospital, Shijiazhuang, Hebei 050050 China; 9 Department of General Surgery, Baoding Central Hospital, Baoding, Hebei 071030 China; 10 General Surgery Department, Hengshui People’s Hospital, Hengshui, Hebei 053099 China; 11 Research Center, Tumor Research Institute of the Fourth Hospital of Hebei Medical University, Shijiazhuang, 050011 China

**Keywords:** Gastric cancer, Lymph node metastases, Liquid biopsy, Transcriptomics panel, Risk stratification assessment

## Abstract

**Background:**

The increasing incidence of early-stage T1 gastric cancer (GC) underscores the need for accurate preoperative risk stratification of lymph node metastasis (LNM). Current pathological assessments often misclassify patients, leading to unnecessary radical surgeries.

**Methods:**

Through analysis of transcriptomic data from public databases and T1 GC tissues, we identified a 4-mRNA panel (SDS, TESMIN, NEB, and GRB14). We developed and validated a Risk Stratification Assessment (RSA) model combining this panel with clinical features using surgical specimens (training cohort: *n* = 218; validation cohort: *n* = 186), gastroscopic biopsies (*n* = 122), and liquid biopsies (training cohort: *n* = 147; validation cohort: *n* = 168).

**Results:**

The RSA model demonstrated excellent predictive accuracy for LNM in surgical specimens (training AUC = 0.890, validation AUC = 0.878), gastroscopic biopsies (AUC = 0.928), and liquid biopsies (training AUC = 0.873, validation AUC = 0.852). This model significantly reduced overtreatment rates from 83.9 to 44.1% in tissue specimens and from 84.4 to 56.0% in liquid biopsies. The 4-mRNA panel showed specificity for T1 GC compared to other gastrointestinal cancers (*P* < 0.001).

**Conclusions:**

We developed and validated a novel liquid biopsy-based RSA model that accurately predicts LNM in T1 GC patients. This non-invasive approach could significantly reduce unnecessary surgical interventions and optimize treatment strategies for high-risk T1 GC patients.

**Supplementary Information:**

The online version contains supplementary material available at 10.1186/s13046-025-03305-x.

## Introduction

The implementation of widespread screening programs and regular evaluations for early gastric cancer (GC) has significantly increased the detection rates of T1 GC by 15–30% in recent years [1–3]. Simultaneously, advancements in endoscopic technologies, including endoscopic submucosal dissection (ESD) and endoscopic mucosal resection (EMR), have enabled curative treatments for patients with T1 GC, who previously required radical surgical interventions [4–6]. These innovations have led to guideline recommendations, including those from the National Comprehensive Cancer Network, establishing ESD as the preferred treatment modality for T1 GC [7–8]. Emerging evidence highlights the potential of ESD as a standalone treatment for T1 GC patients at low risk of lymph node metastasis (LNM). However, radical surgical resection remains the standard for those deemed high-risk [9]. Current risk stratification for LNM predominantly relies on the eCura score, derived from post-endoscopic pathological analysis [10–12]. This approach often misclassifies approximately 70–80% of T1 GC patients as high-risk, despite post-surgical pathological assessments revealing LNM in only 8–16% of cases [13–16]. Consequently, overtreatment with radical surgeries is common, exposing patients to unnecessary costs, complications, and elevated mortality risks associated with extensive surgical procedures.

Accurate preoperative identification of LNM risk is critical for guiding treatment decisions in T1 GC. While ESD is adequate for managing low-risk patients, the reliance on the eCura score frequently results in unnecessary surgical interventions for patients without LNM. This highlights an urgent clinical need for more precise stratification methods to minimize overtreatment, potentially sparing up to 85–95% of patients from radical surgeries [13–16].

Recent studies suggest that mRNA expression patterns can serve as reliable biomarkers, reflecting both physiological and pathological states in GC [17–20]. Differential mRNA expression has been closely associated with gastric carcinogenesis, underscoring its potential for molecular subtyping and risk assessment [21–24]. Recent studies have emphasized the role of gene expression in classifying patients with GC into various molecular subtypes [25–28]. While prior investigations have demonstrated the utility of gene expression profiles in identifying lymphatic involvement in other cancers, such as colorectal and esophageal cancers [29–35], their application in T1 GC remains underexplored. The advent of liquid biopsy technology offers a promising avenue for non-invasive biomarker-based diagnostics. Unlike traditional tissue-based approaches, liquid biopsies enable straightforward, cost-effective, and preoperative risk assessments. This method could transform the clinical management of T1 GC by allowing precise identification of high-risk LNM without the need for invasive procedures.

In this study, we address the critical need for improved LNM risk assessment in T1 GC through the development and validation of a novel liquid biopsy-based transcriptomic panel. By leveraging a comprehensive approach to biomarker discovery, we aim to establish a non-invasive diagnostic tool that accurately identifies high-risk LNM patients. This innovative strategy has the potential to minimize unnecessary radical surgeries, optimize treatment outcomes, and improve the overall quality of care for T1 GC patients.

## Methods and materials

### Biomarker discovery in genome-wide expression profiling datasets

**Fig. 1:**
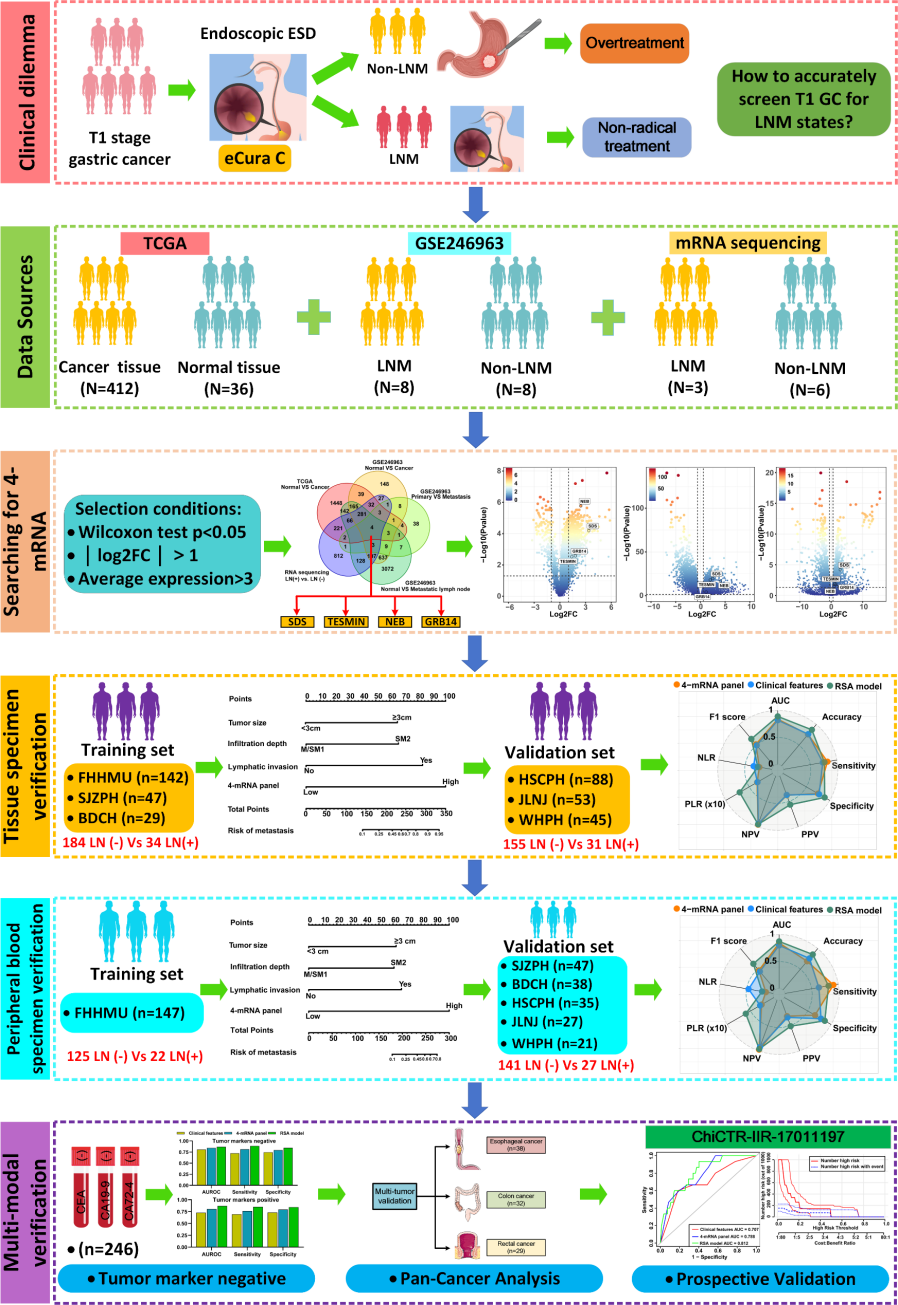
Flowchart of the study design for the discovery and validation of a 4-mRNA panel to predict LNM in T1 GC patients

The workflow of this study, depicted in Fig. 1, involves a systematic biomarker discovery process, followed by validation phases using various sample types, including fresh frozen post-surgical specimens, gastroscopic biopsy specimens, and peripheral blood samples. During the biomarker discovery phase, we selectively used the GSE246963 dataset from the Gene Expression Omnibus (GEO) database (https://www.ncbi.nlm.nih.gov/geo/), adhering to strict screening criteria (Supplementary Fig. 1). Additionally, we analyzed gene expression profiles from patients with GC in The Cancer Genome Atlas (TCGA) database. We also incorporated mRNA sequencing data from three T1-stage gastric cancer tissues with LNM and six without, sourced from the Fourth Hospital of Hebei Medical University (FHHMU).

During the validation phase, we used real-time quantitative polymerase chain reaction (RT-qPCR) to evaluate the expression of these candidate mRNAs in a pilot cohort comprising 28 matched pairs of T1 GC samples with and without LNM, matched based on a 1:1 propensity score. Additionally, we matched 22 pairs of peripheral blood samples from the same period to assess candidate mRNA expression in blood. All samples in the pilot cohort were collected from FHHMU between January and March 2022 and 2024, with the detailed protocol illustrated in Supplementary Fig. 2. The clinicopathological details of these patients are provided in Supplementary Table 1.

### Clinical cohorts for biomarker validation

Initially, this study included 404 fresh-frozen specimens from T1 GC patients for training and validation of biomarkers predictive of LNM. These specimens were sourced from six diagnostic centers across different regions in China. The training cohort comprised 218 patients recruited between January 2010 and January 2024 from the FHHMU, Shijiazhuang People’s Hospital (SJZPH), and Baoding Central Hospital (BDCH). The validation cohort included 186 patients treated between July 2010 and February 2024 at Hengshui People’s Hospital (HSCPH), Jinling Hospital of Nanjing University (JLNJ), and Renmin Hospital of Wuhan University (WHPH). No significant clinical differences were observed between the training and validation cohorts (Supplementary Table 2).

Furthermore, we performed an additional analysis using 122 matched gastroscopic biopsy specimens collected from six diagnostic centers. This analysis complements the study of surgically resected specimens and validates the transition of biomarker detection from larger surgical specimens to smaller gastroscopic biopsies. Detailed clinical characteristics are provided in Supplementary Table 3.

To adapt tissue-based biomarkers for liquid biopsy applications, we retrospectively analyzed a cohort of T1 GC patients. The training cohort consisted of 147 T1 GC patients treated at FHHMU from 2017 to 2020. For the validation cohort, we included serum samples from 168 GC patients collected between 2014 and 2020 across five other institutions (SJZPH, BDCH, LNPCH, JLNJ, and WHPH). Detailed clinical characteristics of both cohorts are summarized in Supplementary Table 4, showing no significant differences between groups. Additionally, we prospectively validated these findings in an independent cohort of 97 T1 GC patients (registration number: ChiCTR-IIR-17011197) recruited at FHHMU from August 2017 to March 2019, with serum samples collected pre- and three months post-radical surgery.

To evaluate the specificity of our candidate mRNAs as biomarkers for gastric cancer, we compared their performance in predicting LNM with that in other endoscopically resectable gastrointestinal cancers, such as esophageal, colon, and rectal cancers. This analysis involved assessing the expression of each gene in the 4-mRNA panel using RT-qPCR on serum samples from gastrointestinal cancer patients. Serum samples from patients with esophageal (*n* = 38), colon (*n* = 32), and rectal cancer (*n* = 29) were collected at FHHMU between 2019 and 2021.

### Inclusion and exclusion criteria

All patients recruited for this study underwent biopsy-confirmed radical surgery for T1-stage GC and were classified as “high-risk” according to the eCure scoring system. High-risk criteria included lesion diameter over 3 cm, positive vertical margins, venous invasion, submucosal invasion depth greater than 500 μm, and positive lymphovascular invasion. Exclusion criteria included patients who had received any antitumor therapy prior to enrollment, those with distant metastasis, cases of residual gastric tumors following partial gastrectomy, non-adenocarcinoma histology, or cases without available serum samples. For all cohorts, recurrence or disease progression was monitored regularly by laboratory tests, endoscopy, and abdominal and pelvic CT according to established gastric cancer treatment guidelines [36].

Tissue specimens were obtained from malignant lesions in surgically resected gastric samples, rapidly frozen in liquid nitrogen, and stored at -80℃. The processing and examination of surgical specimens followed the guidelines of the Chinese Society of Clinical Oncology. Tumor invasion depth (T stage) and lymph node metastasis (N stage) were classified according to the 8th edition of the American Joint Committee on Cancer (AJCC). All procedures adhered to the principles of the Declaration of Helsinki. Written informed consent was obtained from all study participants, and the study received institutional review board approval from all participating institutions.

### RNA extraction and gene expression analysis

Total RNA was isolated from freshly frozen surgical tissues using TRIzol reagent, following the manufacturer’s protocol, and from serum samples using the PAXgene Blood RNA Kit (Qiagen). Detailed RNA isolation, purification, and reverse transcription protocols have been described in our previous studies [37–38]. For gene expression analysis, RT-qPCR was conducted on an Applied Biosystems Real-Time PCR system using the 2^^−ΔΔCT^ method, with GAPDH as the internal control. Assay reproducibility was ensured by implementing control templates, excluding low-quality RNA samples, and performing replicates. PCR primer sequences are provided in Supplementary Table 5.

### Protein-protein Interaction network and pathway analysis

The Protein-Protein Interaction (PPI) network for target genes was constructed using the STRING database (https://string-db.org) and visualized in Cytoscape (https://cytoscape.org), where hub genes were identified through degree analysis. Additionally, the candidate gene list was analyzed for gene set and pathway enrichment using the Enrichr database (https://maayanlab.cloud/Enrichr/) [39].

### Statistical analysis

Statistical analyses were performed using IBM SPSS version 23, R version 3.6.3 and GraphPad Prism version 8.0. The association between mRNA expression and various clinicopathological factors was evaluated using the *X*^2^ test. Paired one-sided t-tests were used to compare gene expression levels in serum samples pre- and post-surgery. Both univariate and multivariate logistic regression analyses, incorporating clinicopathological variables and mRNA classifiers as covariates, were performed; only variables significant in the univariate models were included in the multivariate regression. During the discovery phase, gene expression differences between the LNM and non-LNM groups were analyzed using Wilcoxon rank sum and Bonferroni tests. In the clinical validation phase, gene-based risk scoring was modeled through logistic regression using backward elimination. The performance of these models was assessed using the receiver operating characteristic (ROC) curves and AUC values. AUC values were calculated from ROC curves using the pROC package in R, with ROC curve comparisons performed using the DeLong test. The Youden index in the pROC package was used to determine the optimal cut-off value for the ROC curves. Sensitivity, specificity, positive predictive value (PPV), negative predictive value (NPV), precision, and accuracy for the 4-mRNA panel-based biomarker groups were calculated across all cohorts using the Report ROC package. The results are displayed as confusion matrix plots. Statistical significance was set at *P* < 0.05.

## Results

### Discovery of candidate genes predicting LNM in T1 GC patients

**Fig. 2:**
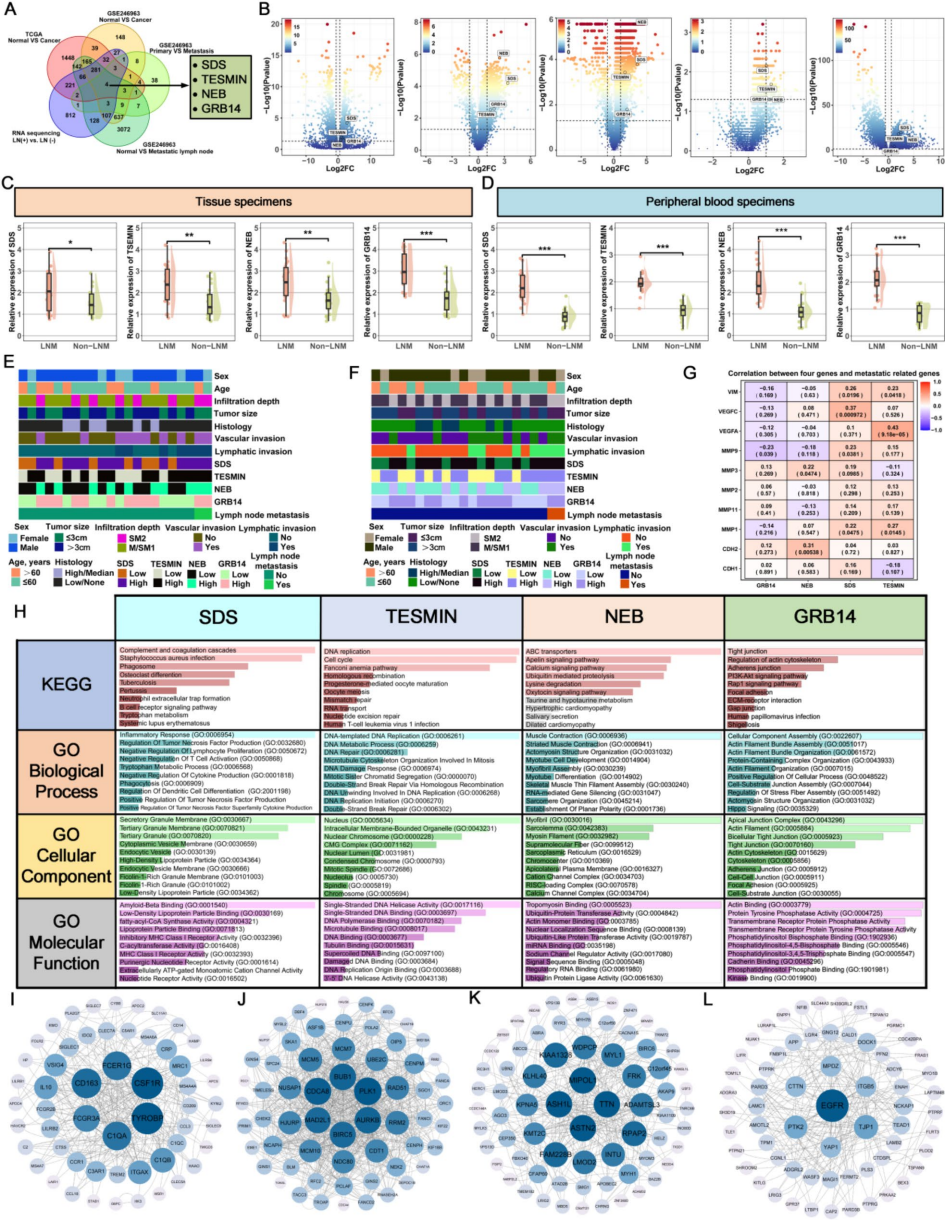
Discovery and Preliminary Validation of LNM Candidate Biomarkers in T1 Gastric Cancer Patients Using Public Databases and Transcriptomic Sequencing Data. (**A**) Through Venn diagram analysis, four candidate mRNAs (SDS, TESMIN, NEB, GRB14) were identified by examining transcriptomic data from the TCGA database (tumor tissues vs. adjacent normal tissues), GEO database (GSE246963), and paired tumor samples from T1 GC patients with and without LNM. (**B**) A volcano plot displays the expression levels of these four candidate genes across the datasets used in the discovery process. (**C**) Expression levels of the four candidate mRNAs were compared in fresh-frozen tumor tissues from 28 matched T1 GC patient pairs with and without LNM, matched by propensity score. (**D**) In peripheral blood samples from 22 matched patient pairs, expression levels of the four candidate mRNAs were also assessed in patients with and without LNM. (**E**) The relationship between the expression levels of the four candidate mRNAs in fresh-frozen tumor samples and clinicopathological characteristics was analyzed. (**F**) Similarly, the relationship between mRNA expression levels in peripheral blood samples and clinicopathological characteristics was evaluated. (**G**) An association heatmap was generated based on the TCGA database to explore the relationship between the four candidate mRNAs and common metastasis-related genes. (**H**) GO and KEGG pathway analyses of the four genes were performed using the Enrichr database. (**I**-**L**) PPI networks for each of the four candidate mRNAs (I: SDS, J: TESMIN, K: NEB, L: GRB14) were constructed using the online STRING database (https://string-db.org)

In this study, we performed an unbiased biomarker discovery process by analyzing transcriptomic data from two GC datasets (TCGA and GSE246963), complemented with mRNA sequencing data from T1 GC tissues with and without LNM. Using differential gene expression analysis (Wilcoxon rank-sum test for GSE246963, *P* < 0.05; EdgeR for TCGA, *P* < 0.05) and correlation analysis (*r* < 0.5), we identified four genes SDS, TESMIN, NEB, and GRB14 that were differentially expressed between LNM and non-LNM patients (Fig. 2A). Volcano plots showed these genes were upregulated in cancer tissues (Fig. 2B). Validation with TCGA data confirmed higher expression in LNM cases (*P* < 0.05) (Supplementary Fig. 3), and pan-cancer analysis showed elevated expression across various cancer types (Supplementary Fig. 4). Further validation in a pilot cohort at FHHMU showed significantly higher expression of these genes in T1 GC tissues with LNM compared to those without LNM (*P* < 0.05) (Fig. 2C-D). Additionally, higher expression levels of these genes were closely associated with poorer clinical characteristics (Fig. 2E-F), and the detailed P values are shown in Supplementary Tables 6–7. Correlation analysis using Timer 2.0 (http://timer.cistrome.org/) indicated positive associations between these genes and VEGFA and VEGFC in metastasis and tube formation (Fig. 2G). Pathway enrichment analysis using Enrichr (KEGG and GO, https://maayanlab.cloud/Enrichr/) and PPI network mapping with STRING and Cytoscape 3.9.1 revealed the potential roles of these genes in GC (Fig. 2H-L). Overall survival analysis of the four candidate mRNAs using Kaplan-Meier plots (https://kmplot.com/analysis/) revealed that high expression status of all four candidate genes was significantly associated with poorer prognosis (Supplementary Fig. 5).

### Validation of surgical resection specimens for 4-mRNA panel predicting LNM in T1 GC patients

**Fig. 3:**
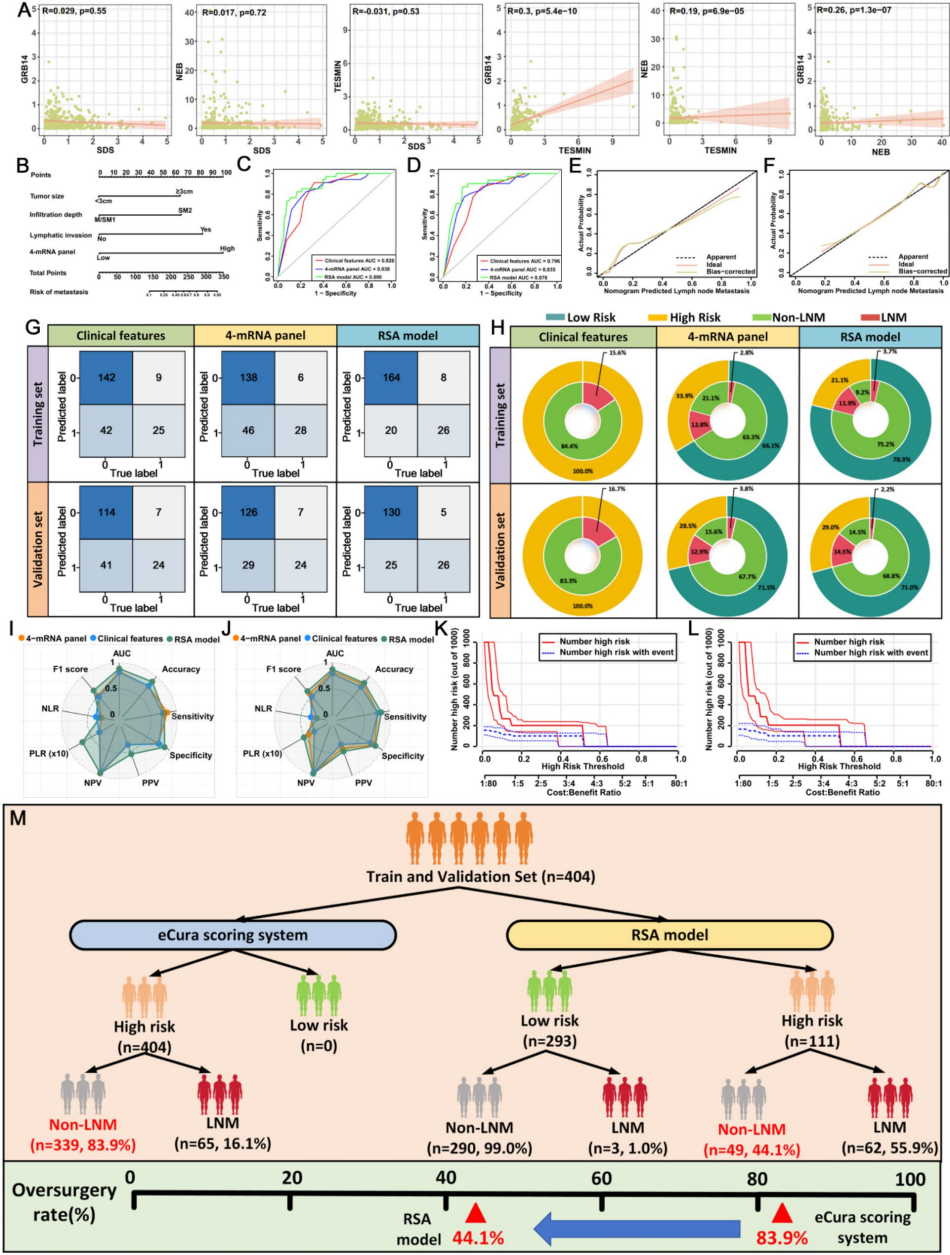
Training and Validation of the 4-mRNA Signature for Predicting LNM in T1 Gastric Cancer Patients Using Fresh-Frozen Tissue Samples. (**A**) Correlation analysis among the four candidate genes. (**B**) Nomogram constructed to predict LNM in T1 GC patients, based on the 4-mRNA signature combined with clinical features. (**C**) ROC curves of various predictive variables within the training dataset. (**D**) ROC curves of different predictive variables within the validation dataset. (**E**) Calibration curve of the RSA model in the training dataset. (**F**) Calibration curve of the RSA model in the validation dataset. (**G**) Confusion matrices for different predictive models in the training and validation datasets. (**H**) Double-layer concentric circle plots displaying clinical benefit for different predictive models in the training and validation datasets. (**I**) Radar chart comparing evaluation metrics of different predictive models in the training dataset. (**J**) Radar chart comparing evaluation metrics of various predictive models in the validation dataset. (**K**) Clinical impact curve of the RSA model for patients in the training dataset. (**L**) Clinical impact curve of the RSA model for patients in the validation dataset. (**M**) Comparative analysis of the eCura scoring system versus the RSA model for identifying LNM, using a combined dataset from the training and validation sets

First, we conducted a correlation analysis of the four candidate mRNA biomarkers and found no significant correlations among them, eliminating the possibility of collinearity (Fig. 3A). Next, we evaluated these biomarkers in a training cohort of T1 GC patients (184 without LNM, 34 with LNM) using RT-qPCR and logistic regression. Each gene was independently associated with LNM risk in T1 GC patients (*P* < 0.05, Supplementary Table 8). ROC curve analysis showed that while individual mRNA biomarkers were effective, the combined 4-mRNA panel significantly enhanced diagnostic performance (AUC = 0.838, sensitivity 82.3%, specificity 75.0%) (Supplementary Fig. 6A-B).

To further improve clinical utility, we developed a Risk Stratification Assessment (RSA) model by combining the 4-mRNA panel (OR = 13.911, 95% CI: 4.585–42.212) with clinical variables, including tumor size (OR = 5.906, 95% CI: 1.673–20.856), depth of infiltration (OR = 5.940, 95% CI: 1.814–19.452), and lymphovascular invasion (OR = 5.935, 95% CI: 1.767–19.935) (Supplementary Table 9). This RSA model was visualized using a nomogram (Fig. 3B). In the training cohort, the RSA model demonstrated excellent predictive accuracy for LNM, achieving an AUC of 0.890, significantly outperforming the clinical model (AUC = 0.820; *P* = 0.036) (Fig. 3C). Calibration curves further validated the RSA model’s reliability in predicting LNM (Fig. 3E). The confusion matrix and radar chart confirmed that the RSA model provided higher sensitivity and specificity than the clinical model alone (Fig. 3G upper panel; Fig. 3I; Supplementary Table 10).

For validation, the RSA model was applied to an independent cohort of 186 T1 GC patients (31 LNM-positive, 155 LNM-negative). It retained high predictive accuracy (AUC = 0.878, sensitivity 83.9%, specificity 83.9%), outperforming both the clinical model and the 4-mRNA panel in LNM detection (Fig. 3D). Calibration curves again verified the model’s reliability in predicting LNM risk (Fig. 3F). The confusion matrix and radar chart confirmed that the RSA model achieved the highest sensitivity and specificity in the validation set as well (Fig. 3G lower panel; Fig. 3J; Supplementary Table 10). Additionally, we selected 26 T2, 19 T3, and 40 T4 GC patients for validation of the RSA model’s ability to predict LNM. The results revealed that the expression of four mRNAs associated with LNM was higher in specimens from T2-T4 stage GC patients with LNM compared to those without (Supplementary Figs. 7–8). Further ROC curve analysis showed the following AUC values: T2 patients AUC = 0.646 (95% CI: 0.400–0.892), T3 patients AUC = 0.608 (95% CI: 0.343–0.873), and T4 patients AUC = 0.640 (95% CI: 0.416–0.864). These findings suggest that the 4-mRNA model may have limited applicability for predicting LNM in T2-T4 stage GC patients (Supplementary Fig. 9).

Clinically, the RSA model significantly reduced overtreatment rates. As shown in Fig. 3H, using traditional clinicopathological criteria, 100% of patients in the training cohort would have been classified as high-risk, resulting in unnecessary radical surgeries for 84.4% of cases (184 of 218). In contrast, the 4-mRNA classifier reduced the high-risk classification rate to 33.9%, with an overtreatment rate of only 21.1%. The RSA model further refined this classification, effectively eliminating overtreatment (9.2% in the training cohort). Similar reductions were observed in the validation cohort, where the RSA model significantly reduced unnecessary surgeries compared to other models. Clinical impact curve analysis demonstrated that the RSA model’s nomogram offered superior net benefit across a broad, practical range of threshold probabilities, indicating substantial predictive value in both training and validation sets (Fig. 3K-L). Overall, the RSA model markedly improved clinical decision-making, reducing overtreatment rates from 83.9 to 44.1% across both cohorts, thus enhancing treatment accuracy and minimizing unnecessary interventions (Fig. 3M). Moreover, compared to the eCura system, the high- and low-risk stratification based on the RSA model was able to distinguish T1 GC patients (Supplementary Fig. 10). The RSA combination model improved the prediction accuracy for recurrence risk compared to the eCura system (AUC = 0.724, 95% CI = 0.640–0.809), with the RSA model achieving an AUC of 0.786 (95% CI = 0.703–0.868) (Supplementary Fig. 12A-C).

### Validation of gastroscopic biopsy specimens for predicting the 4-mRNA panel of LNM in T1GC patients

**Fig. 4:**
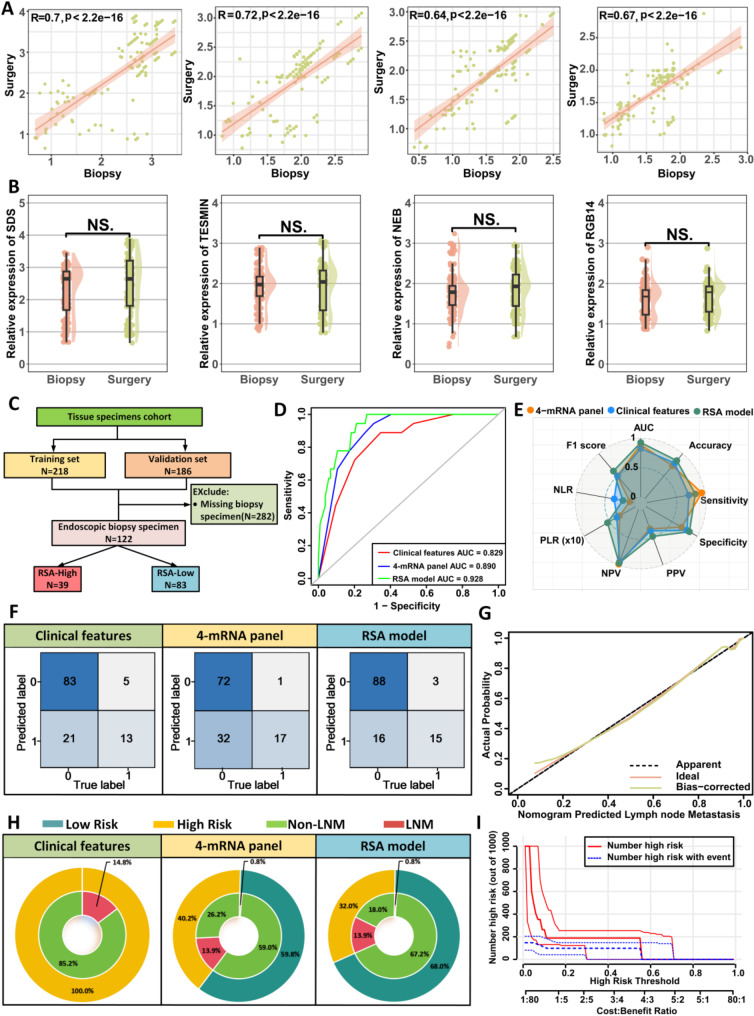
Transcriptome validation stage for identifying LNM in gastroscopic biopsy specimens from patients with T1 GC. (**A**) Correlation analysis of the four mRNAs in gastroscopy biopsy specimens and their paired surgical resection specimens. (**B**) Comparison of expression levels of the four mRNAs in gastroscopy biopsy specimens and paired surgical resection specimens. (**C**) Screening process for the validation set of gastroscopy biopsy specimens. (**D**) ROC curves for various predictor variables in gastroscopy biopsy specimens. (**E**) Radar chart comparing evaluation metrics of different predictive models. (**F**) Confusion matrices of different predictive models. (**G**) Calibration curve of the RSA model in gastroscopy biopsy specimens. (**H**) Double-layer concentric circle plot showing clinical benefits of different predictive models. (**I**) Clinical impact curve of the RSA model within the validation set of gastroscopy biopsy specimens

In addition to the surgically resected specimens from our training cohort, we obtained 122 matched biopsy samples, including 18 cases positive for LNM and 104 negative cases (Fig. 4C). Notably, a significant correlation among four genes was observed in the matched biopsy samples (Fig. 4A). Comparative analysis of gene expression between the matched biopsy and surgical specimens revealed no significant differences in these genes (Fig. 4B). The AUC for detecting LNM using clinical characteristics was 0.829 (95% CI: 0.738–0.919). In contrast, the AUC for the RSA model was 0.928 (95% CI: 0.880–0.977), suggesting that the RSA model is also suitable for preoperative biopsy samples (Fig. 4D; Supplementary Fig. 6C). Additionally, calibration curve analysis further validated the RSA model’s excellent predictive performance (Fig. 4G).

In the biopsy cohort, the RSA model demonstrated the highest sensitivity (83.3%) and specificity (84.6%), outperforming the clinical model (sensitivity: 72.2%; specificity: 79.8%) and the 4-mRNA panel model (sensitivity: 94.4%; specificity: 69.2%) (Fig. 4E–F, Supplementary Table 11). After analyzing the clinical benefits of different models, we found that the RSA model reduced the overtreatment rate from 85.2% to 13.9%, compared to the clinical feature-only model (Fig. 4H). This result highlights the RSA model’s potential to improve clinical decision-making and reduce unnecessary treatments. Furthermore, the clinical impact curve showed that the nomogram provided superior net benefit across a broad, clinically relevant range of threshold probabilities, underscoring the RSA model’s predictive value (Fig. 4I).

### Liquid biopsy specimen validation of a 4-mRNA panel predicting LNM in T1GC patients

**Fig. 5:**
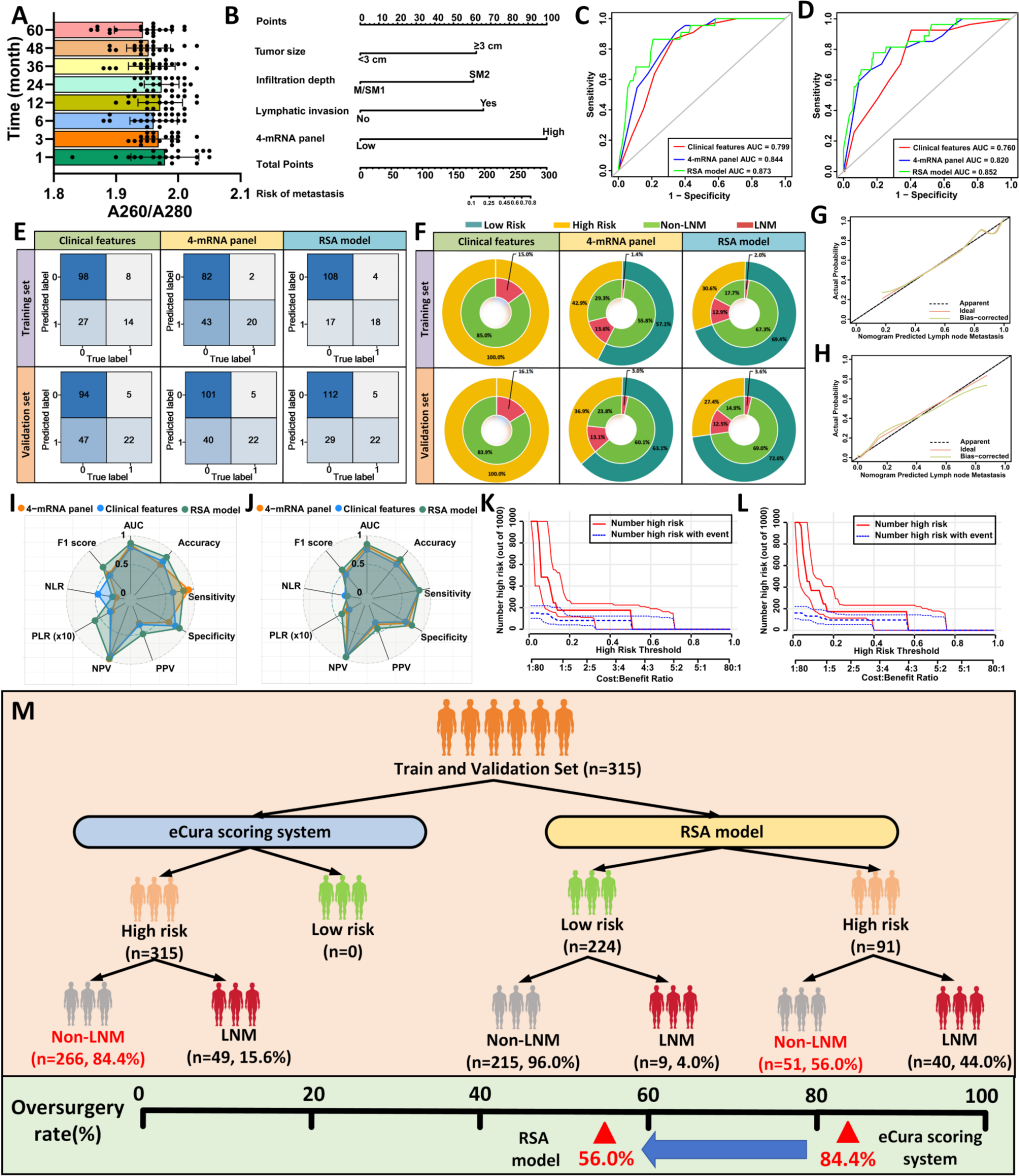
Transcriptome validation phase for identification of LNM in peripheral blood specimens from patients with T1 GC. (**A**) Quality control analysis of peripheral blood specimens at different time points. (**B**) Construction of an LNM prediction nomogram for T1 GC patients based on the 4-mRNA signature combined with clinical features. (**C**) ROC curves of various predictor variables within the training dataset. (**D**) ROC curves of different predictor variables within the validation dataset. (**E**) Confusion matrices of different predictive models in the training and validation datasets. (**F**) Double-layer concentric circle plot illustrating clinical benefits of various predictive models in the training and validation datasets. (**G**) Calibration curve of the RSA model in the training dataset. (**H**) Calibration curve of the RSA model in the validation dataset. (**I**) Radar chart comparing evaluation metrics of different predictive models in the training dataset. (**J**) Radar chart comparing evaluation metrics of various predictive models in the validation dataset. (**K**) Clinical impact curve of the RSA model for patients in the training dataset. (**L**) Clinical impact curve of the RSA model for patients in the validation dataset. (**M**) Comparative analysis of the eCura scoring system versus the RSA model for identifying LNM, using a combined dataset from the training and validation sets

The primary objective of our study was to develop a liquid biopsy-based assay for predicting LNM in T1 GC patients by adapting a tissue-based 4-mRNA biomarker panel into a serum-based test. In a training cohort of 125 LNM-positive and 22 LNM-negative patients, we used RT-qPCR to assess the diagnostic potential of these mRNAs. Initial quality control of peripheral blood samples confirmed normal A260/280 ratios (Fig. 5A). Logistic regression analysis indicated that each mRNA independently predicted LNM risk (all *P* < 0.05, Supplementary Fig. 6D-E, Supplementary Table 8), and multifactor logistic regression was used to construct a predictive nomogram for LNM (Fig. 5B). In the training cohort, the RSA model demonstrated an AUC of 0.873 (95% CI: 0.801–0.945, Fig. 5C), indicating strong predictive power for LNM. Based on a risk probability cutoff derived from the Youden index, T1 GC cases were dichotomized, and confusion matrix and radar plot analyses further supported the model’s predictive accuracy (Fig. 5E-upper; Fig. 5I; Supplementary Table 12). Calibration curve analysis confirmed the model’s high predictive performance (Fig. 5G). Applied to an external validation cohort (141 LNM-negative and 27 LNM-positive T1 GC patients), the RSA model achieved an AUC of 0.852 (95% CI: 0.774–0.930, Fig. 5D) with superior sensitivity (81.5%) and specificity (79.4%) compared to other models (Fig. 5E-lower; Fig. 5J; Supplementary Table 12). Further calibration analysis validated the model’s robust predictive accuracy (Fig. 5H).

The primary aim of our study was to evaluate the clinical utility of the RSA model, which combines a 4-mRNA biomarker panel and clinical features, for non-invasively identifying patients with actual LNM and reducing unnecessary surgeries in others. In the training cohort, only 15.0% of “high-risk” patients (22 of 147) had LNM, while the RSA model reclassified 69.4% as low-risk, reducing the potential overtreatment rate to 17.7% (26 of 147), a significant improvement over the 85.0% rate associated with traditional pathological criteria (Fig. 5F, upper panel). Similar findings were observed in the external validation cohort, where the RSA model markedly reduced overtreatment rates compared to other models (Fig. 5F, lower panel). Furthermore, clinical impact curve analysis across both cohorts supported the RSA model’s superior net benefit across a broad, clinically relevant range of threshold probabilities (Fig. 5K-L). Combined analysis of the training and validation cohorts showed that the RSA model reduced the conventionally assessed overtreatment rate from 84.4% to 56.0% (Fig. 5M), underscoring its effectiveness in clinical applications. In addition, the RSA model-based risk stratification effectively differentiated T1 GC patients, outperforming the eCura system (Supplementary Fig. 11). The RSA combination model improved the prediction accuracy for recurrence risk compared to the eCura system (AUC = 0.700, 95% CI = 0.630–0.771), with the RSA model achieving an AUC of 0.807 (95% CI = 0.744–0.870) (Supplementary Fig. 12D-F).

### 4-mRNA panel shows significant specificity for LNM prediction of T1 GC compared to other gastrointestinal cancers

**Fig. 6:**
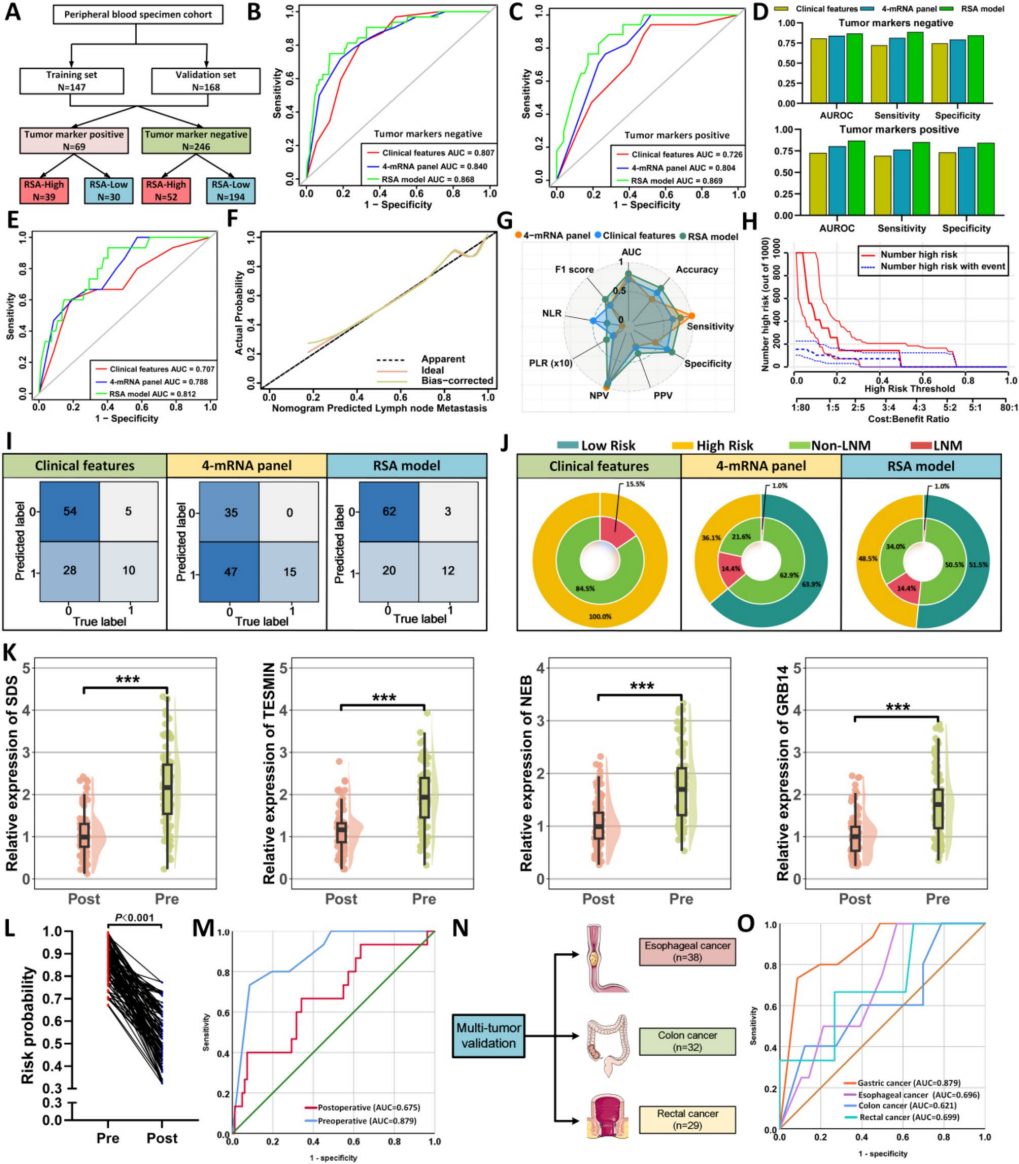
Identification and Prediction of LNM in Patients with Different Peripheral Blood Tumor Marker Status Using a 4-mRNA Signature and Prospective Clinical Validation. (**A**) Distribution of different peripheral blood tumor marker statuses in a new cohort combining the training and validation sets. (**B**-**C**) ROC curves of various predictive models for patients with different peripheral blood tumor marker statuses in the validation set (B, positive; C, negative). (**D**) Comparison of AUC, sensitivity, and specificity for different predictive models in assessing LNM in patients with varying peripheral blood tumor marker statuses. (**E**) ROC curve for LNM prediction in a prospective observational study (ChiCTR-IIR-17011197) using different predictive models. (**F**) Calibration curve of the RSA model in the prospective validation set. (**G**) Radar chart comparing evaluation metrics of different predictive models in the prospective validation set. (**H**) Clinical impact curve of the RSA model within the prospective validation set. (**I**) Confusion matrices for different predictive models in the prospective validation set. (**J**) Double-layer concentric circle plot displaying the clinical benefits of various predictive models in the prospective validation set. (**K**) Comparison of the expression levels of the four mRNAs (I: SDS, J: TESMIN, K: NEB, L: GRB14) in peripheral blood samples taken at baseline and three months post-surgery in the prospective validation set. (**L**) Comparison of LNM risk probabilities based on preoperative and postoperative RSA model formulas constructed from transcriptomic profiles and clinical characteristics in peripheral blood. (**M**) Comparison of ROC curves for LNM prediction based on the 4-mRNA signature in peripheral blood samples before and after surgery. (**N**) Recruitment status of patients with other gastrointestinal malignancies receiving endoscopic treatment. (**O**) ROC curve illustrating the 4-mRNA signature’s performance in predicting LNM in other gastrointestinal malignancies

To assess the specificity of our 4-mRNA panel in predicting LNM in T1 GC patients, we employed a three-pronged validation approach. First, we stratified 315 T1 GC patients in both training and validation cohorts based on peripheral blood tumor markers (CEA, CA19-9, CA72-4), resulting in 69 (21.9%) marker-positive and 246 (78.1%) marker-negative cases (Fig. 6A). Notably, the RSA model (AUC = 0.868, 95% CI: 0.803–0.933; Delong test, *P* < 0.001) outperformed the clinical model (AUC = 0.807, 95% CI: 0.803–0.933) for LNM prediction across both marker-positive and marker-negative cohorts (Fig. 6B–D).

The second approach involved prospective serum samples from a cohort (ChiCTR-IIR-17011197), collected pre-surgery (baseline) and at three months post-surgery (follow-up). ROC curve analysis demonstrated that the RSA model was the most accurate predictor, with an AUC of 0.812 (95% CI: 0.706–0.918), sensitivity of 80.0%, and specificity of 75.6% (*P* < 0.001; Fig. 6E). Calibration curve analysis closely matched the ideal result (Fig. 6F), and confusion matrix and radar plot analyses further confirmed the RSA model’s superior performance over both the clinical model (AUC = 0.707, sensitivity 66.7%, specificity 65.9%) and the 4-mRNA model alone (AUC = 0.788, sensitivity 100.0%, specificity 42.7%) (Fig. 6G, I, Supplementary Fig. 6F; Supplementary Table 13). Clinical benefit analysis demonstrated that the RSA model significantly reduced the high overtreatment rate from 84.5% to 14.4%, highlighting its potential for improved clinical decision-making and fewer unnecessary interventions (Fig. 6H, J). Further analysis of postoperative samples revealed significantly reduced levels of all four mRNAs (Fig. 6K) and a marked decrease in LNM risk probability (*P* < 0.001; Fig. 6L). ROC analysis showed a substantial drop in predictive accuracy for LNM post-surgery, with AUC declining to 0.675, emphasizing the biomarkers’ preoperative specificity (Delong test, *P* < 0.001; Fig. 6M).

In the third approach, we extended the analysis to assess the 4-mRNA panel’s diagnostic performance in other early gastrointestinal cancers (all T1 stage), including esophageal (*n* = 38), colon (*n* = 32), and rectal (*n* = 29) cancers (Fig. 6N). The 4-mRNA panel achieved significantly higher diagnostic accuracy for LNM in GC (AUC = 0.879) than in other cancers (esophageal: AUC = 0.621; colon: AUC = 0.696; rectal: AUC = 0.699; Fig. 6O). DeLong’s test confirmed the GC-specificity with statistically significant differences when compared to esophageal (*P* = 0.002), colon (*P* = 0.001), and rectal cancers (*P* = 0.001). Overall, these findings highlight the high specificity and clinical applicability of the 4-mRNA panel as a non-invasive blood-based biomarker, particularly well-suited for predicting LNM in T1 GC patients.

### Biological characteristics and immune infiltration

**Fig. 7:**
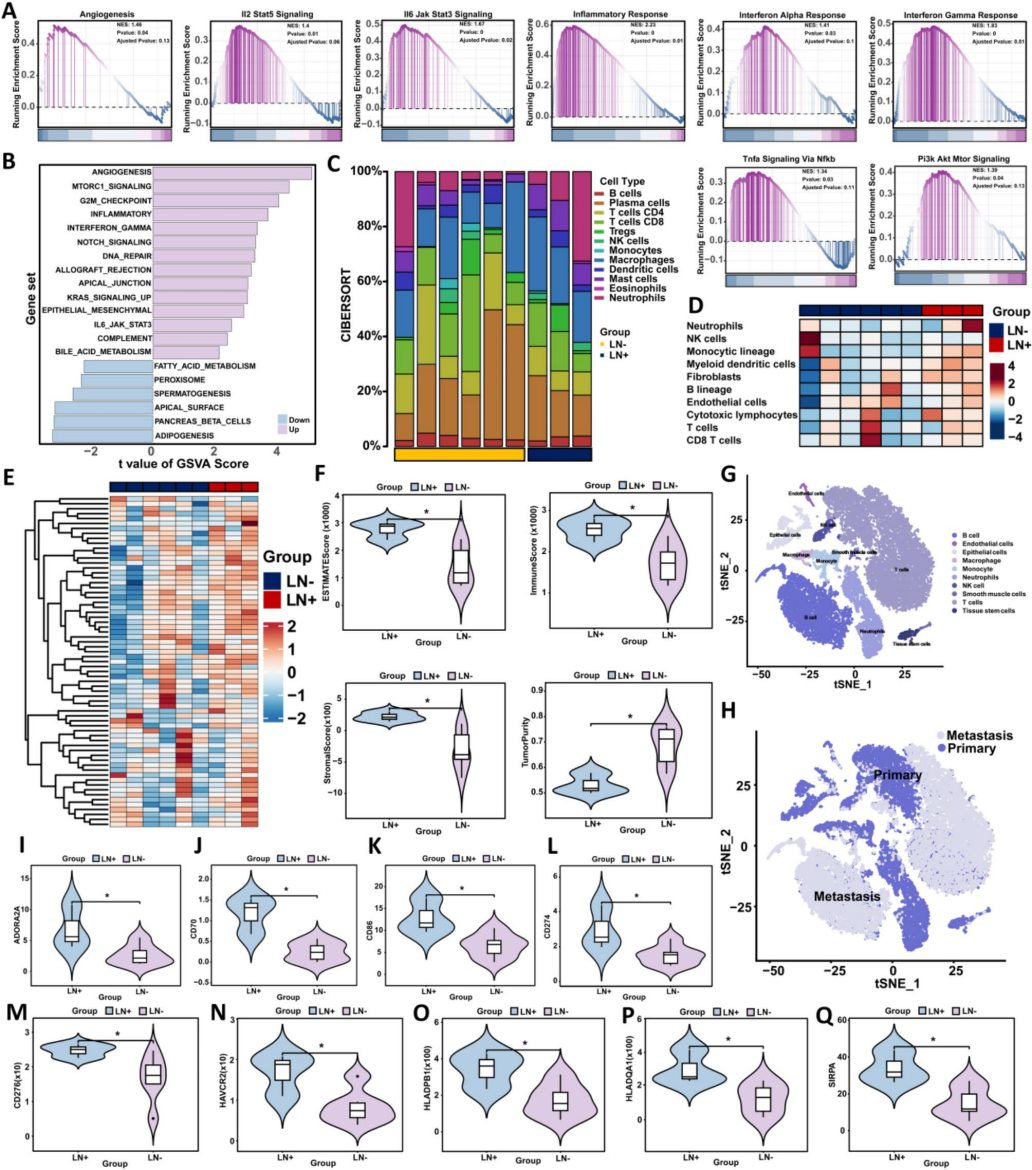
**Biological characteristics and immune infiltration in LNM and Non-LNM groups.** (**A**-**B**) GSEA (**A**) and GSVA (**B**) enrichment analysis results from RNA sequencing data of gastric cancer samples, comparing LNM and Non-LNM groups. (**C**) Scores of combined cell types derived from the CIBERSORT algorithm, illustrating the proportional diversity among features. (**D**) Relative abundance of each immune cell calculated using the MCPcounter algorithm, displayed in a heat map. (**E**) Heat map showing expression levels of immune checkpoint genes in gastric cancer patients with LNM compared to those without LNM. (**F**) Violin plots illustrating differences in tumor purity, immune score, ESTIMATE score, and stromal score between gastric cancer patients with and without LNM. (**G**-**H**) t-SNE plots depicting cell type (**G**) and metastasis type (**H**) derived from single-cell data of gastric cancer patients. (**I**-**Q**) Violin plots displaying differences in immune checkpoint gene expression between gastric cancer patients with LNM and those without LNM

To explore the immunological characterization of this feature, we performed GSEA functional enrichment analysis using RNA sequencing data of gastric cancer samples (LNM + vs. Non-LNM), and the results showed that tumor progression and immune regulation-related pathways such as Agiogenesis, PI3K AKT MTOR signaling, Inflammatory response, IL2 STAT5 signaling, Interferon α response, Interferon γ response, and TNFα pathway were significantly upregulated in the lymph node metastasis group (Fig. 7A). In addition, the same trend was shown in further GSVA enrichment analysis (Fig. 7B). Since cell types vary with local signaling networks and drive cellular activities within tumors, we investigated whether cell states and multicellular communities differ between different features. We calculated the relative abundance of each immune cell in tumor tissue using two algorithms, CIRBERSORT and MCPcounter. In terms of cell state, the results showed a trend of higher abundance of immune cells in patients with lymph node metastasis (Fig. 7C-D).

Next, we focused on the characterization of immune infiltration in the local immune signaling environment. We calculated the immune infiltration score of each sample by ESTIMATE analysis, and the results showed that the stromal, immune and ESTIMATE scores of patients in the LNM group were significantly higher than those in the Non-LNM group (Fig. 7F). Further immune checkpoint analysis results showed that most immune-related targets were expressed more highly in gastric cancer patients with lymph nodes (Fig. 7E, I-Q). These results indicate that immunotherapy has a potential effect on gastric cancer populations with lymph nodes. To further explore the biological functions that affect this feature, we used single-cell transcriptomes to reveal the potential role of related genes in the immune microenvironment. We obtained 10 cell subsets (Fig. 7G-H) by screening, dimensionality reduction, clustering and cell grouping of single-cell data, and showed the expression of 4 genes in the immune microenvironment. The results showed that SDS, TESMIN and NEB were all expressed in T cells and B cells (Supplementary Fig. 13).

## Discussion

Our study addresses the limitations of currently used clinicopathological risk profiles in identifying LNM in “high-risk” subgroups of patients with T1 GC, where LNM presence is a crucial factor for additional surgery following curative endoscopic treatment. To the best of our knowledge, this is the first study to employ transcriptomics-based liquid biopsy for predicting LNM in patients with pathologically high-risk T1 GC. Our findings indicate that transcriptomic liquid biopsies using peripheral blood can accurately estimate the preoperative risk, offering significant clinical advantages for more effective risk stratification in LNM detection. This approach could substantially reduce the current overuse of surgical intervention in these patients. By accurately identifying high-risk T1 GC patients who truly have LNM and sparing others from unnecessary treatments, this method has the potential to decrease patient complications, lessen physician workload, and reduce associated healthcare costs.

In this study, we performed biomarker discovery by integrating data from two major public databases and six pairs of matched specimens subjected to mRNA sequencing. This approach enabled us to identify four mRNAs that are closely associated with LNM in patients with T1 GC. These mRNAs were significantly upregulated in GC patients compared to those in adjacent normal mucosal tissues. We used the expression data of these mRNAs from surgically resected specimens, along with clinical features, to construct the RSA model for LNM. This model was validated in an external cohort from multiple centers, demonstrating that the diagnostic accuracy of the RSA model for LNM (AUC = 0.890) significantly surpassed that of the existing clinical risk models (AUC = 0.820 [training] and 0.796 [validation]). Although all patients in our study were considered high-risk for LNM and underwent radical surgery, postoperative pathological analysis indicated that only 16.1% of patients (65 of 404, including both the training and validation cohorts) required this treatment, while 83.9% underwent unnecessary surgery. In contrast, our RSA model indicated overtreatment in only 44.1% of cases, showing its superior effectiveness in LNM identification. Furthermore, we conducted re-validation using paired surgical biopsy specimens to evaluate whether the 4-mRNA panel could provide greater accuracy in identifying LNM in T1 GC, particularly in preoperative biopsies. The findings revealed that the RSA model, based on the 4-mRNA panel and clinical characteristics, achieved an AUC of 0.928 (95%CI: 0.880–0.977), indicating enhanced diagnostic performance even in preoperative biopsy specimens.

Numerous studies have shown the potential of endoscopic ultrasonography and abdominal CT in diagnosing LNM in patients with T1 GC; however, their diagnostic accuracy for LNM is often deemed insufficient [40–43]. Given that current clinical guidelines classify the presence of LNM as a critical factor in identifying high-risk T1 GC patients, there is a clear need to develop robust LNM biomarkers for pretreatment assessment, which could significantly transform clinical decision-making [7–8]. In our study, we converted these tissue-based markers into blood-based liquid biopsy assays and assessed their efficacy in predicting LNM in multiple independent serum-based clinical cohorts of patients with T1 GC. Through extensive training and validation of serum markers, we developed RSA model based on a 4-mRNA panel and clinically relevant variables. This model reliably identifies T1 GC patients with LNM. The successful validation of the RSA model in predicting LNM in pre-treatment serum samples highlights its clinical importance in refining therapeutic approaches for patients with T1 GC, particularly those with confirmed LNM. Previous research has primarily focused on single specimen validation, often limited to single gene expression, without comprehensive sample-level validation for LNM diagnosis [44–48]. This approach can significantly influence the selection of appropriate therapeutic strategies. The pre-operative application of our transcriptomic biomarkers as a robust, straightforward, and cost-effective assay aimed to minimize unnecessary surgical interventions, thereby reducing post-operative complications, surgery-related mortality, and the overall economic burden associated with such invasive procedures.

Furthermore, recent studies have addressed survival and recurrence risks in patients with early-stage GC. Hatta et al. emphasized that not all patients who do not meet the curative criteria for ESD require radical surgery, suggesting that some high-risk early gastric cancer patients may benefit from a more tailored approach to treatment [49]. Similarly, Suzuki et al. reported clinical outcomes of early GC patients after noncurative ESD and highlighted the importance of distinguishing between those who truly require further treatment and those who do not, thereby helping to avoid overtreatment [50]. These findings underscore the need for improved predictive tools, such as our RSA model, which can more accurately assess recurrence and metastasis risk, thus informing clinical decisions and potentially reducing unnecessary surgeries.

### Limitations

Our study has several potential limitations owing to its retrospective design, which may have introduced selection bias. First, the relatively small sample size, particularly the limited number of patients with LNM, might have influenced the outcomes of our model construction. Therefore, future prospective clinical trials with larger patient cohorts are essential to validate the diagnostic accuracy of our constructed RSA model. Second, our training and validation cohorts consisted exclusively of Chinese patients who exhibited specific clinicopathological characteristics. These characteristics might differ in patient populations from other countries, suggesting the need for cross-national studies with larger sample sizes to evaluate biomarker performance comprehensively. Such studies would help to translate these biomarkers into routine clinical practice and enhance the generalizability of our findings. Morover, we acknowledge that due to limited follow-up, our study currently lacks long-term survival data. However, we have outlined a long-term follow-up research plan to further assess the relationship between biomarker-based models and clinical outcomes. Lastly, while our risk stratification model incorporated mRNA and clinical factors, previous reports have highlighted the association of high expression of common clinical markers, such as HER2 and PDL1, and the presence of DNA mutations with LNM risk. Considering that fewer factors are generally more feasible for clinical application, future research should explore additional factors such as HER2, PDL1 markers, or DNA mutation status to determine whether these factors enhance the diagnostic accuracy for LNM detection. Despite these limitations, our study provides significant evidence for the detection of LNM in T1 GC patients, potentially contributing to the development of robust molecular biomarkers for the risk assessment and management of these fatal malignancies.

## Conclusions

In conclusion, our study successfully identified and developed a novel risk stratification model that utilized liquid biopsy assays for the detection of LNM. This model enables more reliable and accurate identification of high-risk patients with T1 GC. Subject to validation in future prospective studies, our findings underscore the potential clinical significance of this model for optimizing the selection of patients with high-risk T1 GC. By doing so, it could substantially reduce the burden of unnecessary medical procedures, lower the associated costs, and enhance the overall management of patients with pathologically high-risk T1 GC.

## Electronic supplementary material

Below is the link to the electronic supplementary material.

Supplementary Material 1

## Data Availability

The participant data with identifiers used to support the findings of this study were supplied by Qun Zhao under license, and thus cannot be made freely available. The requests for access to these data should be made to Qun Zhao, zhaoqun@hebmu.edu.cn.

## References

[1] Shen M, Xia R, Luo Z, Zeng H, Wei W, Zhuang G, et al. The long-term population impact of endoscopic screening programmes on disease burdens of gastric cancer in China: a mathematical modelling study. J Theor Biol. 2020;484:109996. 10.1016/j.jtbi.2019.109996. 31491497 10.1016/j.jtbi.2019.109996

[2] Wang FH, Zhang XT, Tang L, Wu Q, Cai MY, Li YF, et al. The Chinese Society of Clinical Oncology (CSCO): clinical guidelines for the diagnosis and treatment of gastric cancer, 2023. Cancer Commun (Lond). 2024;44(1):127–72. 10.1002/cac2.12516. 38160327 10.1002/cac2.12516 PMC10794017

[3] Xin Y, Zhang Q, Liu X, Li B, Mao T, Li X. Application of artificial intelligence in endoscopic gastrointestinal tumors. Front Oncol. 2023;13:1239788. 10.3389/fonc.2023.1239788. 38144533 10.3389/fonc.2023.1239788 PMC10747923

[4] Panarese A. Endoscopic resection for early gastric cancer: towards a global understanding. World J Gastroenterol. 2022;28(13):1377–9. 10.3748/wjg.v28.i13.1377. 35645546 10.3748/wjg.v28.i13.1377 PMC9099183

[5] Draganov PV, Wang AY, Othman MO, Fukami N. AGA Institute Clinical Practice Update: endoscopic submucosal dissection in the United States. Clin Gastroenterol Hepatol. 2019;17(1):16–e251. 10.1016/j.cgh.2018.07.041. 30077787 10.1016/j.cgh.2018.07.041

[6] Pimentel-Nunes P, Libânio D, Bastiaansen BAJ, Bhandari P, Bisschops R, Bourke MJ, et al. Endoscopic submucosal dissection for superficial gastrointestinal lesions: European Society of Gastrointestinal Endoscopy (ESGE) Guideline - Update 2022. Endoscopy. 2022;54(6):591–622. 10.1055/a-1811-7025. 35523224 10.1055/a-1811-7025

[7] ASGE standards of practice committee, Forbes N, Elhanafi SE, Al-Haddad MA, Thosani NC, Draganov PV, et al. The American Society for Gastrointestinal Endoscopy guideline on endoscopic submucosal dissection for the management of early esophageal and gastric cancers: Summary and recommendations. Gastrointest Endosc. 2023;98(3):271–84. 10.1016/j.gie.2023.03.015. 37498266 10.1016/j.gie.2023.03.015

[8] Libânio D, Pimentel-Nunes P, Bastiaansen B, Bisschops R, Bourke MJ, Deprez PH, et al. Endoscopic submucosal dissection techniques and technology: European Society of Gastrointestinal Endoscopy (ESGE) Technical Review. Endoscopy. 2023;55(4):361–89. 10.1055/a-2031-0874. 36882090 10.1055/a-2031-0874

[9] Vos EL, Nakauchi M, Gönen M, Castellanos JA, Biondi A, Coit DG, et al. Risk of Lymph Node Metastasis in T1b gastric Cancer: an International Comprehensive Analysis from the Global Gastric Group (G3) alliance. Ann Surg. 2023;277(2):e339–45. 10.1097/SLA.0000000000005332. 34913904 10.1097/SLA.0000000000005332 PMC9192823

[10] Hatta W, Gotoda T, Oyama T, Kawata N, Takahashi A, Yoshifuku Y, et al. A Scoring System to Stratify Curability after Endoscopic Submucosal dissection for early gastric Cancer: eCura system. Am J Gastroenterol. 2017;112(6):874–81. 10.1038/ajg.2017.95. 28397873 10.1038/ajg.2017.95

[11] Morais R, Libanio D, Dinis Ribeiro M, Ferreira A, Barreiro P, Bourke MJ, et al. Predicting residual neoplasia after a non-curative gastric ESD: validation and modification of the eCura system in the western setting: the W-eCura score. Gut. 2023;73(1):105–17. 10.1136/gutjnl-2023-330804. 37666656 10.1136/gutjnl-2023-330804

[12] Jin CQ, Zhao J, Ding XY, Yu LL, Ye GL, Zhu XJ, Shen JW, Yang Y, Jin B, Zhang CL, Lv B. Clinical outcomes and risk factors of non-curative endoscopic submucosal dissection for early gastric cancer: a retrospective multicenter study in Zhejiang, China. Front Oncol. 2023;13:1225702. 10.3389/fonc.2023.1225702. 37854682 10.3389/fonc.2023.1225702 PMC10580067

[13] Hatta W, Gotoda T, Oyama T, Kawata N, Takahashi A, Yoshifuku Y, et al. Is the eCura system useful for selecting patients who require radical surgery after noncurative endoscopic submucosal dissection for early gastric cancer? A comparative study. Gastric Cancer. 2018;21(3):481–9. 10.1007/s10120-017-0769-7. 28983696 10.1007/s10120-017-0769-7

[14] Lee S, Kim SG, Cho SJ. Decision to perform additional surgery after non-curative endoscopic submucosal dissection for gastric cancer based on the risk of lymph node metastasis: a long-term follow-up study. Surg Endosc. 2023;37(10):7738–48. 10.1007/s00464-023-10324-2. 37567980 10.1007/s00464-023-10324-2

[15] Niwa H, Ozawa R, Kurahashi Y, Kumamoto T, Nakanishi Y, Okumura K, et al. The eCura system as a novel indicator for the necessity of salvage surgery after non-curative ESD for gastric cancer: a case-control study. PLoS ONE. 2018;13(10):e0204039. 10.1371/journal.pone.0204039. 30273388 10.1371/journal.pone.0204039 PMC6166923

[16] Tian YT, Ma FH, Wang GQ, Zhang YM, Dou LZ, Xie YB, et al. Additional laparoscopic gastrectomy after noncurative endoscopic submucosal dissection for early gastric cancer: a single-center experience. World J Gastroenterol. 2019;25(29):3996–4006. 10.3748/wjg.v25.i29.3996. 31413533 10.3748/wjg.v25.i29.3996 PMC6689811

[17] Serra O, Galán M, Ginesta MM, Calvo M, Sala N, Salazar R. Comparison and applicability of molecular classifications for gastric cancer. Cancer Treat Rev. 2019;77:29–34. 10.1016/j.ctrv.2019.05.005. 31195213 10.1016/j.ctrv.2019.05.005

[18] Tirino G, Pompella L, Petrillo A, Laterza MM, Pappalardo A, Caterino M, et al. What’s New in Gastric Cancer: the therapeutic implications of Molecular classifications and Future perspectives. Int J Mol Sci. 2018;19(9):2659. 10.3390/ijms19092659. 30205505 10.3390/ijms19092659 PMC6165492

[19] Chen D, Cheung H, Lau HC, Yu J, Wong CC. N6-Methyladenosine RNA-Binding protein YTHDF1 in gastrointestinal cancers: function, molecular mechanism and clinical implication. Cancers (Basel). 2022;14(14):3489. 10.3390/cancers14143489. 35884552 10.3390/cancers14143489 PMC9320224

[20] Ding P, Wu J, Wu H, Li T, Niu X, Yang P, Guo H, Tian Y, He J, Yang J, Gu R, Zhang L, Meng N, Li X, Guo Z, Meng L, Zhao Q. Transcriptomics-based Liquid Biopsy for early detection of recurrence in locally advanced gastric Cancer. Adv Sci (Weinh). 2024;11(47):e2406276. 10.1002/advs.202406276. 39556695 10.1002/advs.202406276 PMC11653671

[21] Guo T, Tang XH, Gao XY, Zhou Y, Jin B, Deng ZQ, et al. A liquid biopsy signature of circulating exosome-derived mRNAs, miRNAs, and lncRNAs predict therapeutic efficacy to neoadjuvant chemotherapy in patients with advanced gastric cancer. Mol Cancer. 2022;21(1):216. 10.1186/s12943-022-01684-9. 36510184 10.1186/s12943-022-01684-9 PMC9743536

[22] Qiu MZ, Li ZH, Zhou ZW, Li YH, Wang ZQ, Wang FH, et al. Detection of carcinoembryonic antigen messenger RNA in blood using quantitative real-time reverse transcriptase-polymerase chain reaction to predict recurrence of gastric adenocarcinoma. J Transl Med. 2010;8:107. 10.1186/1479-5876-8-107. 21040522 10.1186/1479-5876-8-107 PMC2989934

[23] Kang Y, Zhang J, Sun P, Shang J. Circulating cell-free human telomerase reverse transcriptase mRNA in plasma and its potential diagnostic and prognostic value for gastric cancer. Int J Clin Oncol. 2013;18(3):478–86. 10.1007/s10147-012-0405-9. 22527847 10.1007/s10147-012-0405-9

[24] Arigami T, Uenosono Y, Hirata M, Yanagita S, Ishigami S, Natsugoe S. B7-H3 expression in gastric cancer: a novel molecular blood marker for detecting circulating tumor cells. Cancer Sci. 2011;102(5):1019–24. 10.1111/j.1349-7006.2011.01877.x. 21251161 10.1111/j.1349-7006.2011.01877.x

[25] Shi W, Wang Y, Xu C, Li Y, Ge S, Bai B, et al. Multilevel proteomic analyses reveal molecular diversity between diffuse-type and intestinal-type gastric cancer. Nat Commun. 2023;14(1):835. 10.1038/s41467-023-35797-6. 36788224 10.1038/s41467-023-35797-6 PMC9929250

[26] Ho SWT, Sheng T, Xing M, Ooi WF, Xu C, Sundar R, et al. Regulatory enhancer profiling of mesenchymal-type gastric cancer reveals subtype-specific epigenomic landscapes and targetable vulnerabilities. Gut. 2023;72(2):226–41. 10.1136/gutjnl-2021-326483. 35817555 10.1136/gutjnl-2021-326483

[27] Chida K, Kawazoe A, Suzuki T, Kawazu M, Ueno T, Takenouchi K, et al. Transcriptomic profiling of MSI-H/dMMR gastrointestinal tumors to identify determinants of responsiveness to Anti-PD-1 therapy. Clin Cancer Res. 2022;28(10):2110–7. 10.1158/1078-0432.CCR-22-0041. 35254400 10.1158/1078-0432.CCR-22-0041 PMC9365358

[28] Wang J, Qin D, Tao Z, Wang B, Xie Y, Wang Y, et al. Identification of cuproptosis-related subtypes, construction of a prognosis model, and tumor microenvironment landscape in gastric cancer. Front Immunol. 2022;13:1056932. 10.3389/fimmu.2022.1056932. 36479114 10.3389/fimmu.2022.1056932 PMC9719959

[29] Zhou H, Zhu L, Song J, Wang G, Li P, Li W, et al. Liquid biopsy at the frontier of detection, prognosis and progression monitoring in colorectal cancer. Mol Cancer. 2022;21(1):86. 10.1186/s12943-022-01556-2. 35337361 10.1186/s12943-022-01556-2 PMC8951719

[30] Raza A, Khan AQ, Inchakalody VP, Mestiri S, Yoosuf ZSKM, Bedhiafi T, et al. Dynamic liquid biopsy components as predictive and prognostic biomarkers in colorectal cancer. J Exp Clin Cancer Res. 2022;41(1):99. 10.1186/s13046-022-02318-0. 35292091 10.1186/s13046-022-02318-0 PMC8922757

[31] Nakamura K, Hernández G, Sharma GG, Wada Y, Banwait JK, González N, et al. Liquid biopsy signature for detection of patients with early onset colorectal cancer. Gastroenterology. 2022;163(5):1242–e12512. 10.1053/j.gastro.2022.06.089. 35850198 10.1053/j.gastro.2022.06.089 PMC9613521

[32] Miyazaki K, Wada Y, Okuno K, Murano T, Morine Y, Ikemoto T, et al. Exosome-based liquid biopsy signature for pre-operative identification of lymph node metastasis in patients with pathological high-risk T1 colorectal cancer. Mol Cancer. 2023;22(1):2. 10.1186/s12943-022-01685-8. 36609320 10.1186/s12943-022-01685-8 PMC9817247

[33] Kandimalla R, Ozawa T, Gao F, Wang X, Goel A, T1 Colorectal Cancer Study Group. Gene expression signatures in surgical issues and endoscopic biopsies identified high-risk T1 colorectal cancers. Gastroenterology. 2019;156(8):2338–e23413. 10.1053/j.gastro.2019.02.027. 30797795 10.1053/j.gastro.2019.02.027 PMC6538250

[34] Ozawa T, Kandimalla R, Gao F, Nozawa H, Hata K, Nagata H, et al. A MicroRNA signature Associated with metastasis of T1 colorectal cancers to Lymph Nodes. Gastroenterology. 2018;154(4):844–e8487. 10.1053/j.gastro.2017.11.275. 29199088 10.1053/j.gastro.2017.11.275 PMC5847452

[35] Xue L, Zhao Z, Wang M, Ma L, Lin H, Wang S, et al. A liquid biopsy signature predicts lymph node metastases in T1 esophageal squamous cell carcinoma: implications for precision treatment strategies. Br J Cancer. 2022;127(11):2052–9. 10.1038/s41416-022-01997-y. 36207607 10.1038/s41416-022-01997-y PMC9681756

[36] Ajani JA, D’Amico TA, Bentrem DJ, Chao J, Cooke D, Corvera C, et al. Gastric Cancer, Version 2.2022, NCCN Clinical Practice guidelines in Oncology. J Natl Compr Canc Netw. 2022;20(2):167–92. 10.6004/jnccn.2022.0008. 35130500 10.6004/jnccn.2022.0008

[37] Ding P, Wu H, Wu J, Li T, He J, Ju Y, Liu Y, Li F, Deng H, Gu R, Zhang L, Guo H, Tian Y, Yang P, Meng N, Li X, Guo Z, Meng L, Zhao Q. N6-methyladenosine modified circPAK2 promotes lymph node metastasis via targeting IGF2BPs/VEGFA signaling in gastric cancer. Oncogene. 2024;43(34):2548–63. 10.1038/s41388-024-03099-w. 39014193 10.1038/s41388-024-03099-w

[38] Ding P, Wu H, Wu J, Li T, Gu R, Zhang L, Yang P, Guo H, Tian Y, He J, Yang J, Meng N, Li X, Meng L, Zhao Q. Transcriptomics-based liquid biopsy panel for early non-invasive identification of peritoneal recurrence and micrometastasis in locally advanced gastric cancer. J Exp Clin Cancer Res. 2024;43(1):181. 10.1186/s13046-024-03098-5. 38937855 10.1186/s13046-024-03098-5 PMC11212226

[39] Xie Z, Bailey A, Kuleshov MV, Clarke DJB, Evangelista JE, Jenkins SL, et al. Gene set knowledge discovery with enrichment. Curr Protocols. 2021;1(3):e90. 10.1002/cpz1.90. 10.1002/cpz1.90 PMC8152575 33780170

[40] Mocellin S, Pasquali S. Diagnostic accuracy of endoscopic ultrasonography (EUS) for the preoperative locoregional staging of primary gastric cancer. Cochrane Database Syst Rev. 2015;2015(2):CD009944. 10.1002/14651858.CD009944.pub2. 25914908 10.1002/14651858.CD009944.pub2 PMC6465120

[41] Chen C, Song YL, Wu ZY, Chen J, Zhang Y, Chen L. Diagnostic value of conventional endoscopic ultrasound for lymph node metastasis in upper gastrointestinal neoplasia: a meta-analysis. World J Gastroenterol. 2023;29(30):4685–700. 10.3748/wjg.v29.i30.4685. 37662859 10.3748/wjg.v29.i30.4685 PMC10472901

[42] Pierantoni C, Lisotti A, Fusaroli P. Prediction of the risk of Lymph Node metastases in early gastric Cancer: contrast-enhanced harmonic endoscopic Ultrasonography May help. Gut Liver. 2021;15(6):940–1. 10.5009/gnl210122. 34140430 10.5009/gnl210122 PMC8593506

[43] Sun Z, Li J, Wang T, Xie Z, Jin L, Hu S. Predicting perigastric lymph node metastasis in gastric cancer with CT perfusion imaging: a prospective analysis. Eur J Radiol. 2020;122:108753. 10.1016/j.ejrad.2019.108753. 31794892 10.1016/j.ejrad.2019.108753

[44] An HJ, Lee JS, Yang JW, Kim MH, Na JM, Song DH. *RAB27A* and *RAB27B* expression levels may predict lymph node metastasis and survival in patients with gastric cancer. Cancer Genomics Proteom. 2022 Sep-Oct;19(5):606–13. 10.21873/cgp.20345. 10.21873/cgp.20345 PMC9353728 35985682

[45] You X, Wang Y, Wu J, Liu Q, Chen D, Tang D, et al. Aberrant cytokeratin 20 mRNA expression in Peripheral Blood and Lymph Nodes indicates Micrometastasis and poor prognosis in patients with gastric carcinoma. Technol Cancer Res Treat. 2019;18:1533033819832856. 10.1177/1533033819832856. 30827194 10.1177/1533033819832856 PMC6856971

[46] Song Z, Zhao W, Cao D, Zhang J, Chen S. Elementary screening of lymph node metastasis-related genes in gastric cancer based on the co-expression network of messenger RNA, microRNA, and long non-coding RNA. Braz J Med Biol Res. 2018;51(4):e6685. 10.1590/1414-431x20176685. 29489999 10.1590/1414-431X20176685 PMC5856436

[47] Miyachi K, Sasaki K, Onodera S, Taguchi T, Nagamachi M, Kaneko H, et al. Correlation between survivin mRNA expression and lymph node metastasis in gastric cancer. Gastric Cancer. 2003;6(4):217–24. 10.1007/s10120-003-0255-2. 14716515 10.1007/s10120-003-0255-2

[48] Zhang J, Liu F, Yang Y, Yu N, Weng X, Yang Y, et al. Integrated DNA and RNA sequencing reveals early drivers involved in metastasis of gastric cancer. Cell Death Dis. 2022;13(4):392. 10.1038/s41419-022-04838-1. 35449126 10.1038/s41419-022-04838-1 PMC9023472

[49] Hatta W, Gotoda T, Oyama T, Kawata N, Takahashi A, Yoshifuku Y, Hoteya S, Nakamura K, Hirano M, Esaki M, Matsuda M, Ohnita K, Shimoda R, Yoshida M, Dohi O, Takada J, Tanaka K, Yamada S, Tsuji T, Ito H, Hayashi Y, Nakamura T, Shimosegawa T. Is radical surgery necessary in all patients who do not meet the curative criteria for endoscopic submucosal dissection in early gastric cancer? A multi-center retrospective study in Japan. J Gastroenterol. 2017;52(2):175–84. 10.1007/s00535-016-1210-4. 27098174 10.1007/s00535-016-1210-4

[50] Suzuki H, Oda I, Abe S, Sekiguchi M, Nonaka S, Yoshinaga S, Saito Y, Fukagawa T, Katai H. Clinical outcomes of early gastric cancer patients after noncurative endoscopic submucosal dissection in a large consecutive patient series. Gastric Cancer. 2017;20(4):679–89. 10.1007/s10120-016-0651-z. 27722825 10.1007/s10120-016-0651-z

