# Supplementary material for: Preoperative liquid biopsy transcriptomic panel for risk assessment of lymph node metastasis in T1 gastric cancer
Source: J Exp Clin Cancer Res. 2025 Feb 7;44:43. doi: 10.1186/s13046-025-03305-x (PMC11804050; doi:10.1186/s13046-025-03305-x)
Supplement: Supplementary file 1 — Supplementary Material 1 [file 13046_2025_3305_MOESM1_ESM.docx]

**Supplementary Figures**

**
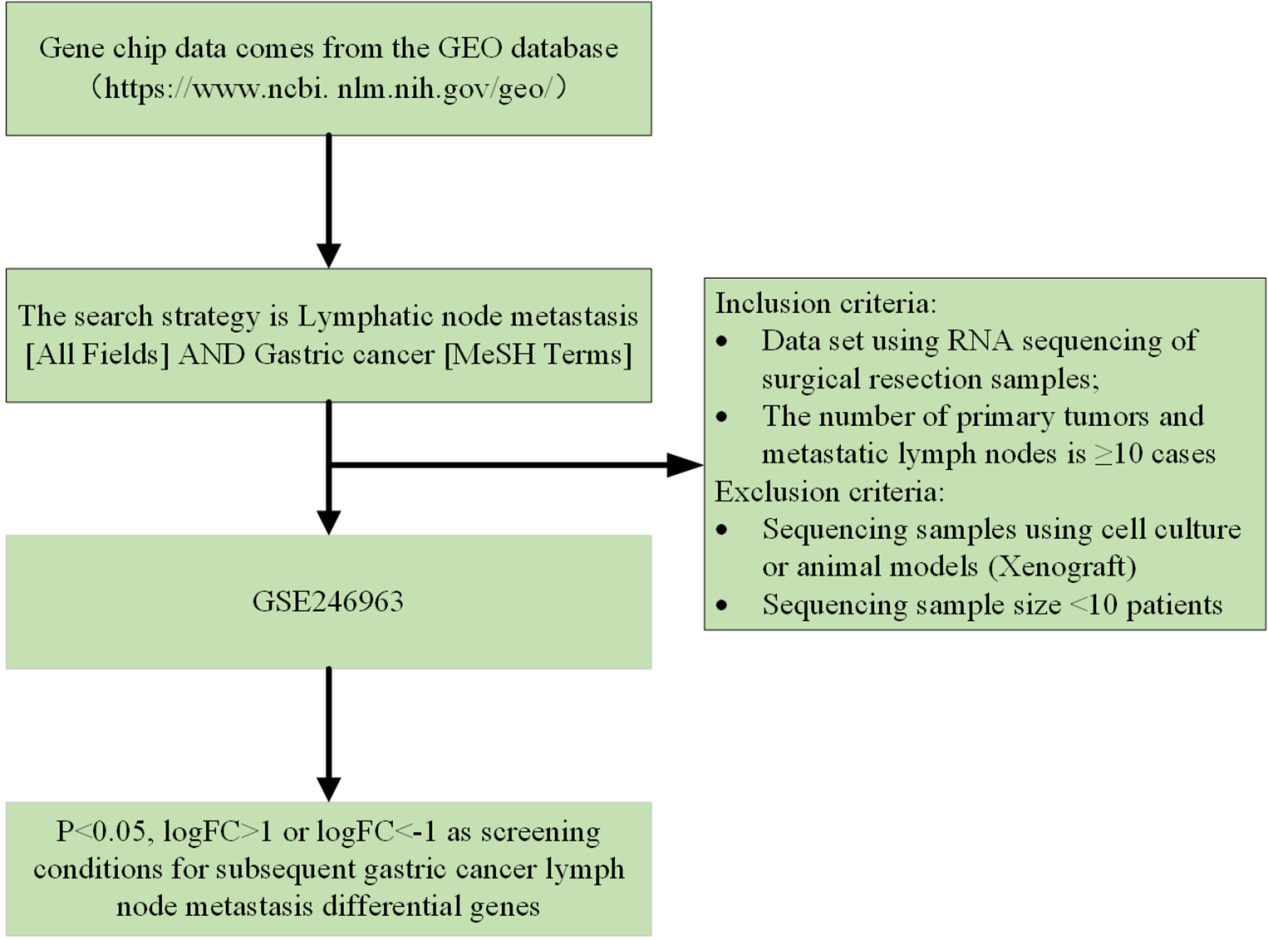
**

**Supplementary Figure 1. The screening and selection process of candidate biomarker datasets in the GEO database.**

**
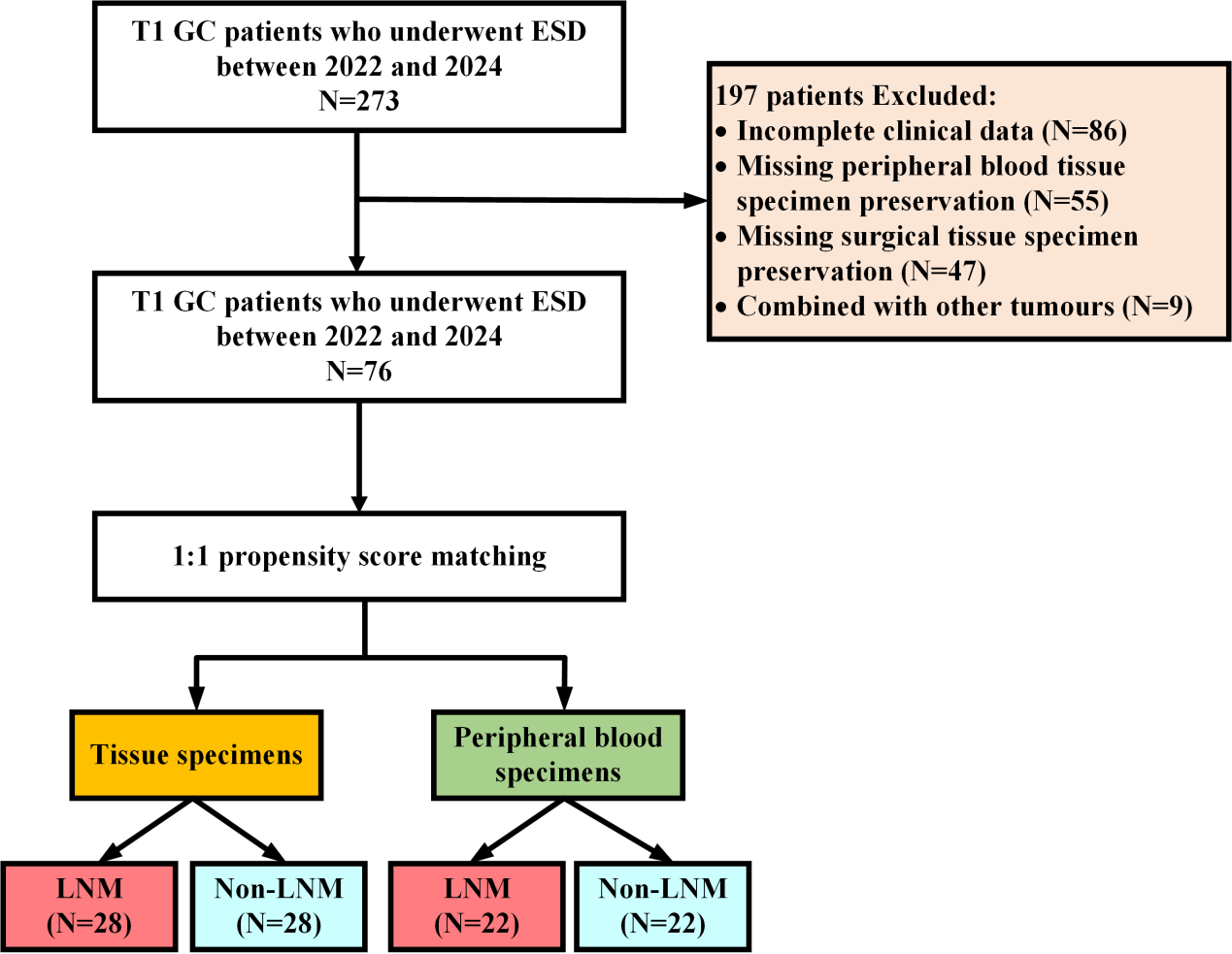
**

**Supplementary Figure 2. Flowchart describing propensity score matching of fresh frozen tissue specimens and peripheral blood specimens in the pilot cohort.**

**
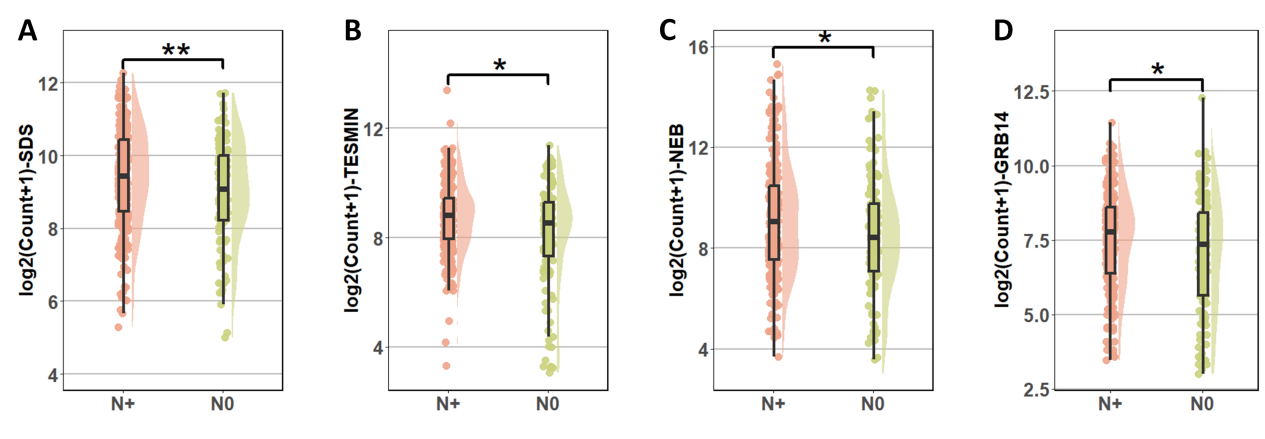
**

**Supplementary Figure 3. Comparison of the expression of four candidate genes in cancer tissue specimens of gastric cancer patients with lymph node metastasis and without lymph nodes in the TCGA database. (A) SDS; (B) TESMIN; (C) NEB; (D) GRB14.**

**
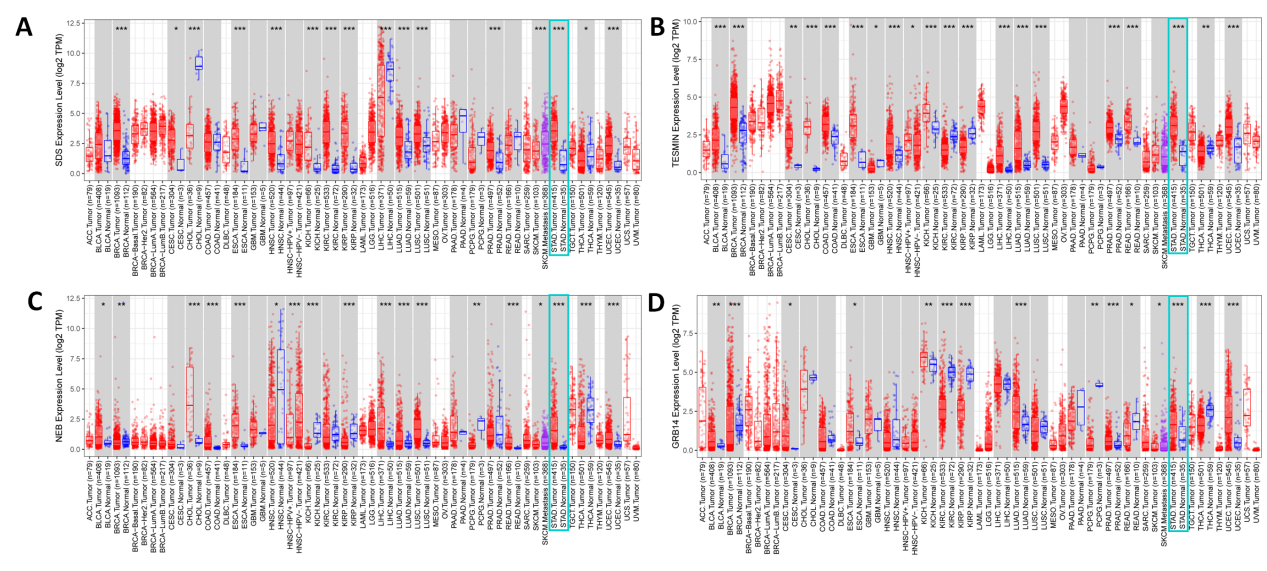
**

**Supplementary Figure 4. Comparison of the expression of four candidate genes in cancer tissues and adjacent tissues of different tumor types. (A) SDS; (B) TESMIN; (C) NEB; (D) GRB14.**

**
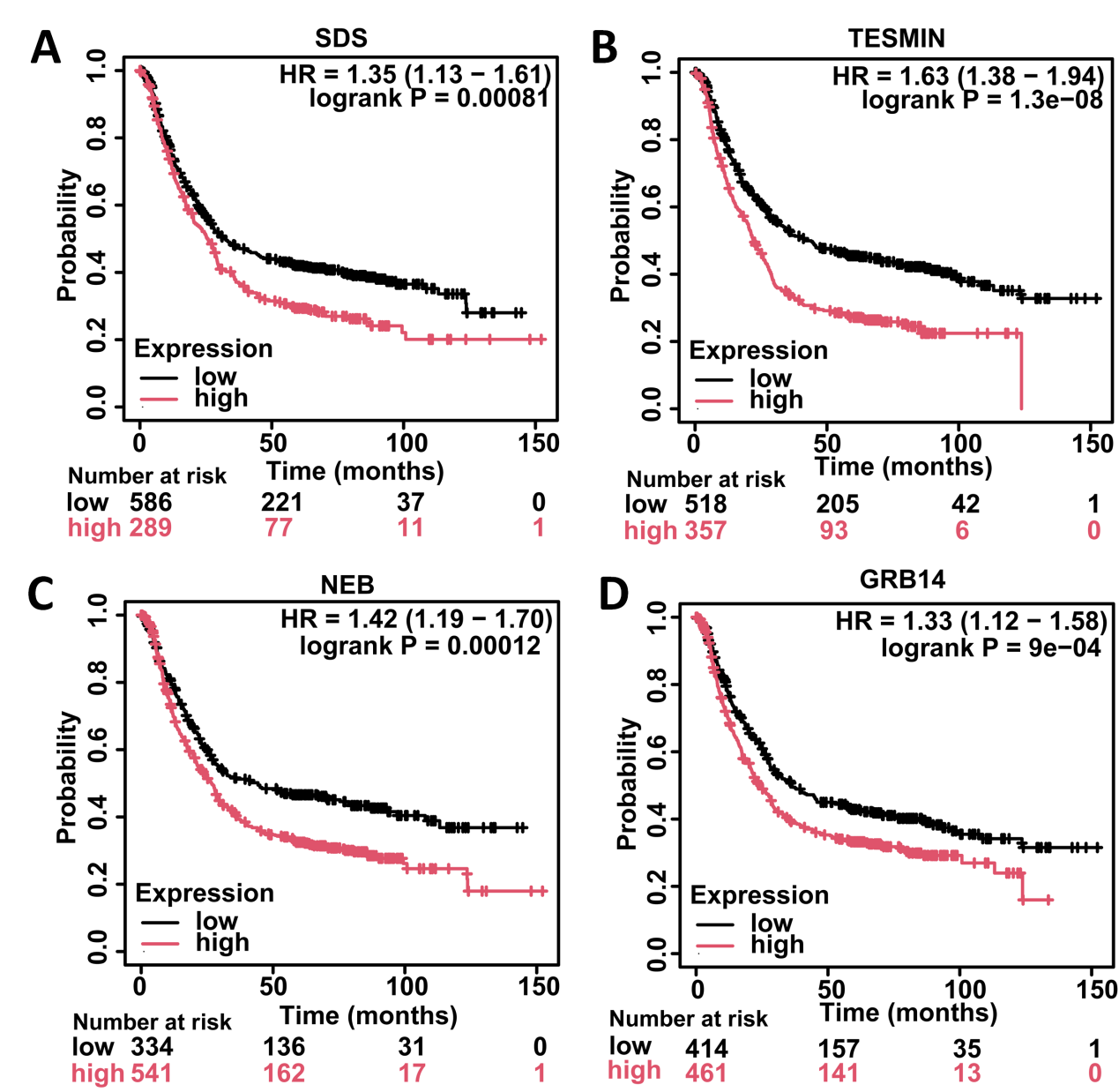
**

**Supplementary Figure 5. Overall survival analysis of the four candidate mRNAs was performed using Kaplan-Meier plots, available at [https://kmplot.com/analysis/]. (A) SDS; (B) TESMIN; (C) NEB; (D) GRB14.**

**
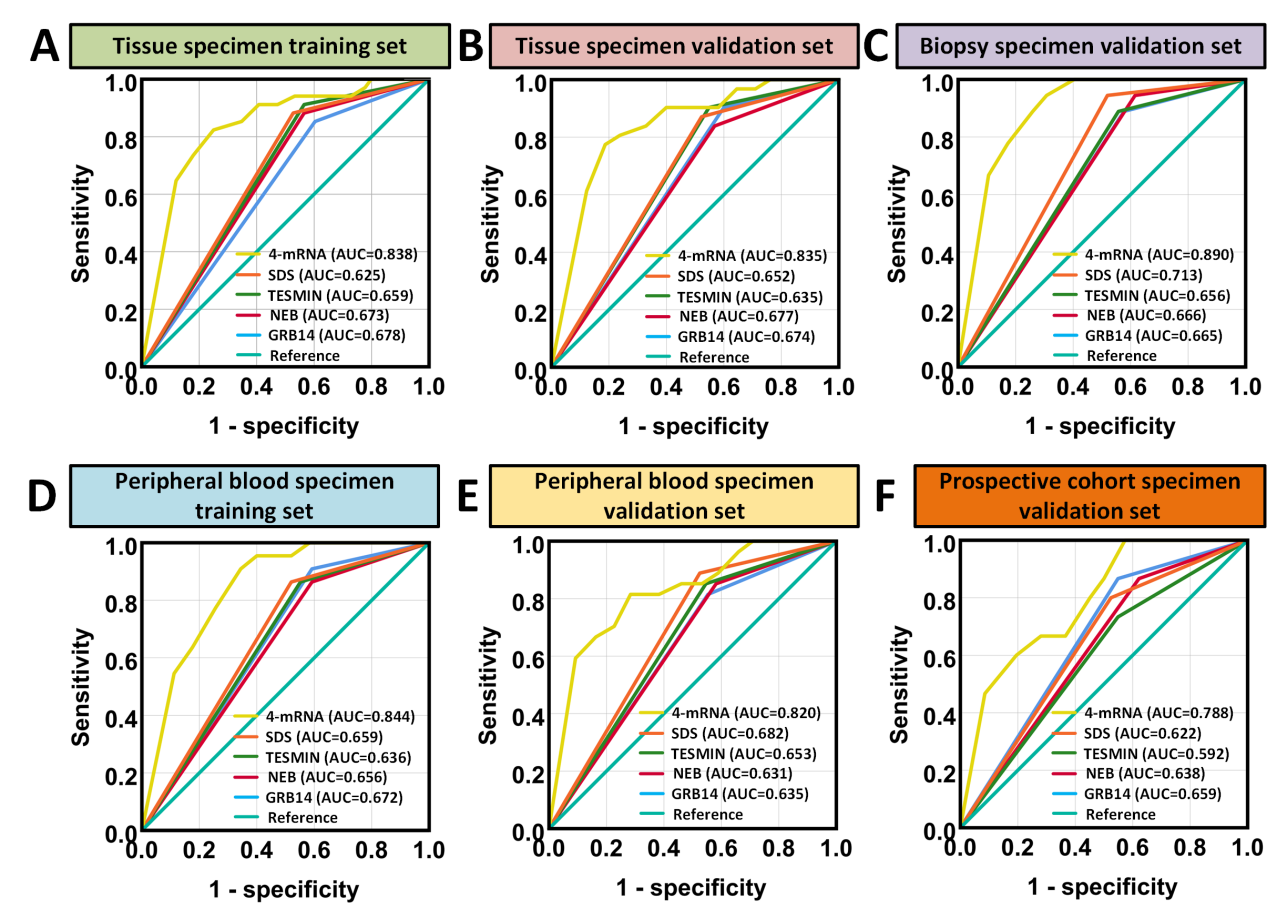
**

**Supplementary Figure 6. Comparison of ROC curves of four candidate mRNAs and the 4-mRNA panel in combination. (A-B) ROC curves of different predictors in the tissue specimen training set (A) and validation set (B). (C) ROC curves of different predictors in the gastroscopy biopsy specimen validation set. (D-E) ROC curves of different predictors in the peripheral blood specimen training set (D) and validation set (E). (F) ROC curves of different predictors in the peripheral blood specimen validation set in the prospective cohort.**

**
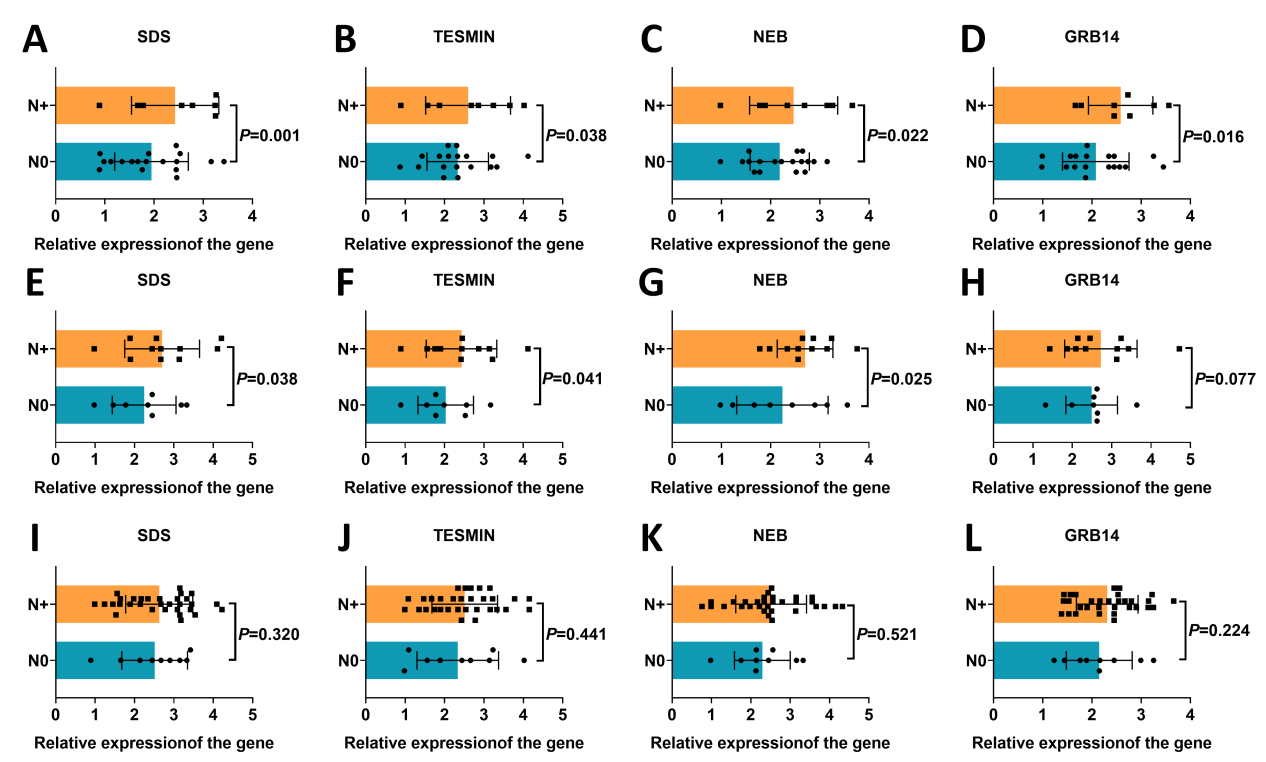
**

**Supplementary Figure 7. Differential analysis of 4-mRNA gene expression in T2-T4 GC patients in the LNM group versus the non-LNM group.** **A-D: T2 GC patients; E-H: T3 GC patients; I-L: T4 GC patients.**


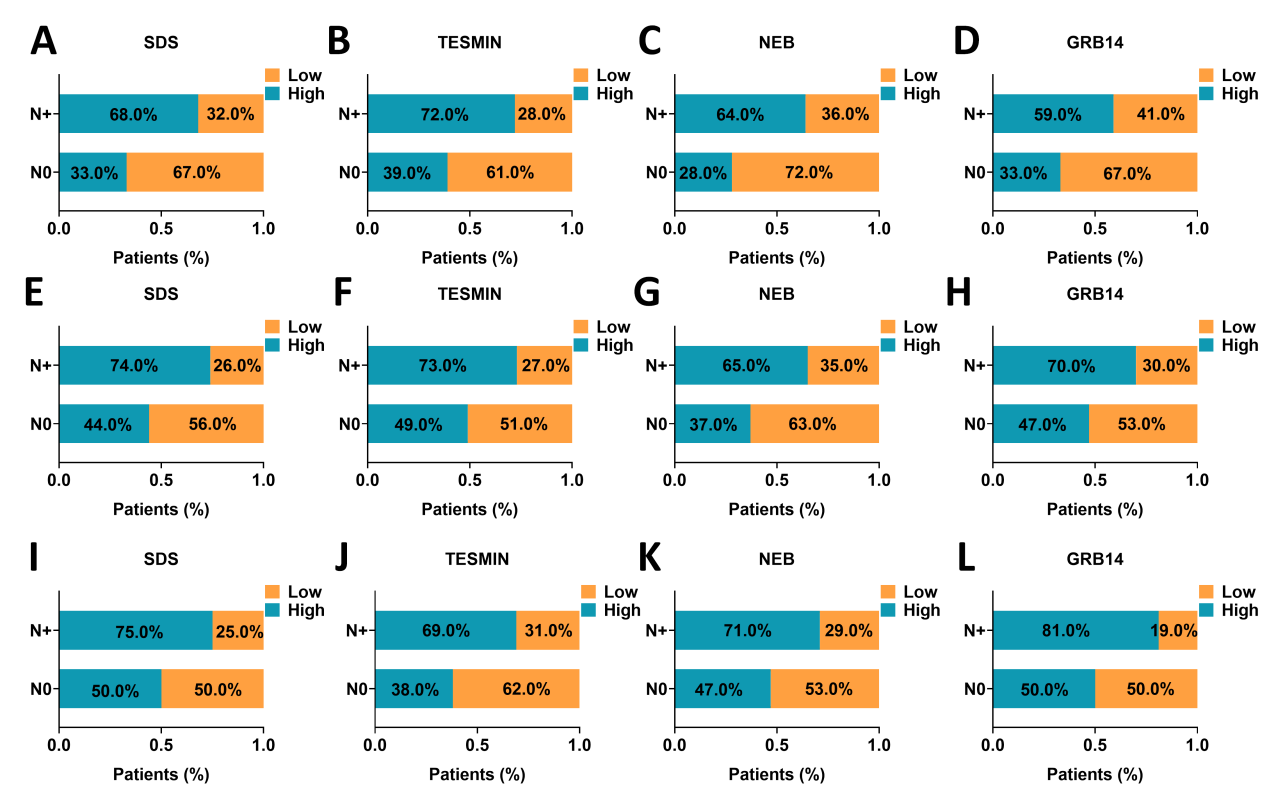


**Supplementary Figure 8**. **Analysis of the percentage of high and low expression of 4-mRNA genes in the LNM group versus the non-LNM group of GC patients with stage T2-T4. A-D: GC patients with stage T2; E-H: GC patients with stage T3; I-L: GC patients with stage T4. All high and low expression subgroups were divided by the median value of each gene in different groups.**


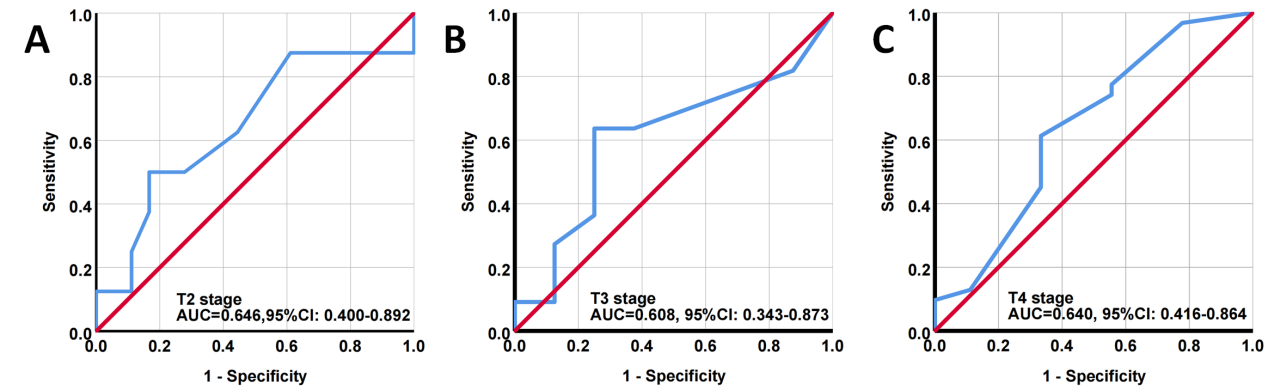


**Supplementary Figure 9**. **ROC curve analysis of 4-mRNA in predicting LNM in patients with T2-T4 stage GC. A: T2 stage GC; B: T3 stage GC; C: T4 stage GC.**


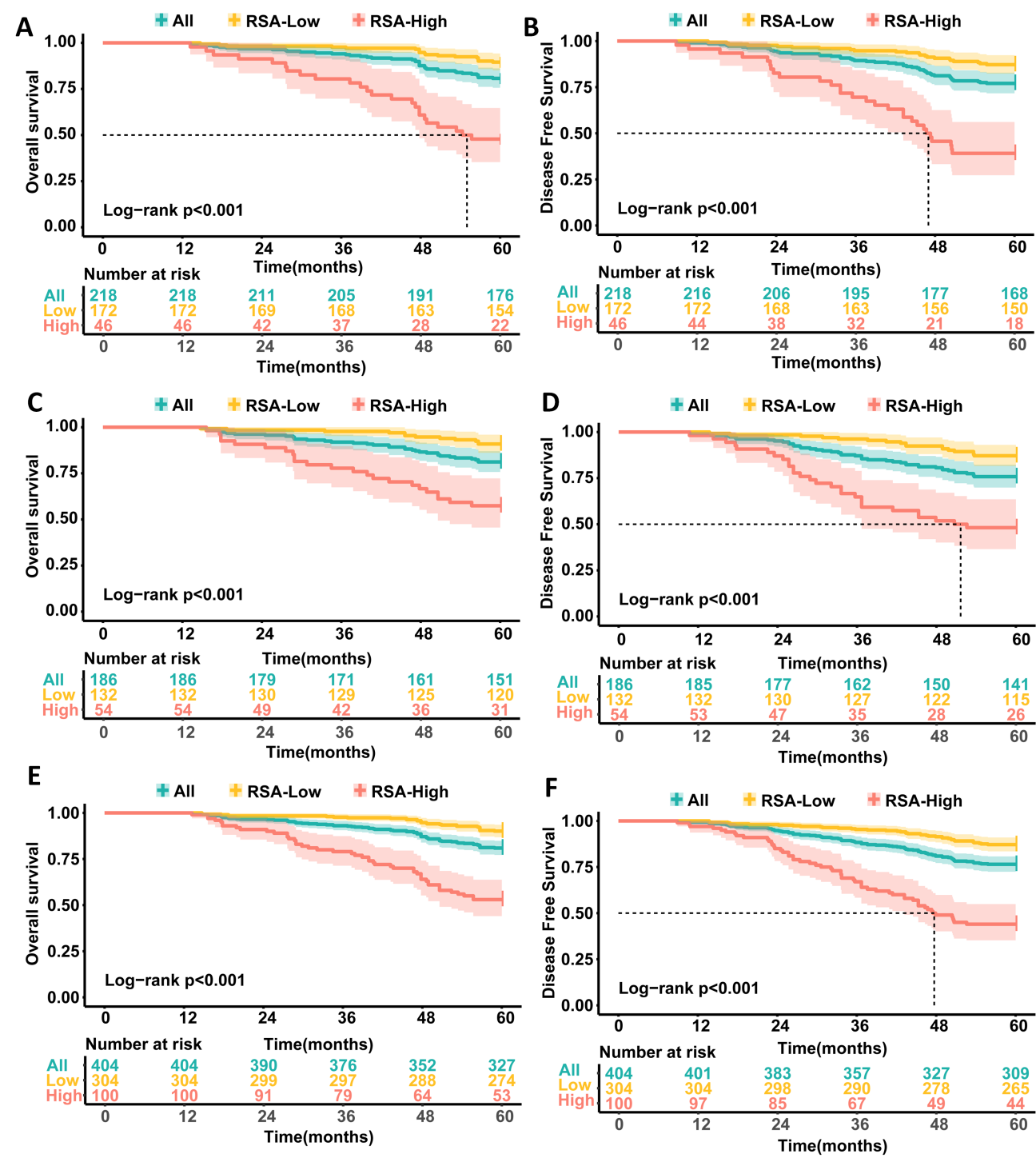


**Supplementary Figure 10. Survival curves based on low- and high-risk stratification using the eCura system and RSA model across different datasets of tissue samples. A. 5-year overall survival (OS) curve for the tissue sample training cohort; B. 5-year disease-free survival (DFS) curve for the tissue sample training cohort; C. 5-year OS curve for the tissue sample validation cohort; D. 5-year DFS curve for the tissue sample validation cohort. E. 5-year OS curve for the combined dataset (training + validation cohorts) of tissue samples; F. 5-year DFS curve for the combined dataset (training + validation cohorts) of tissue samples.**


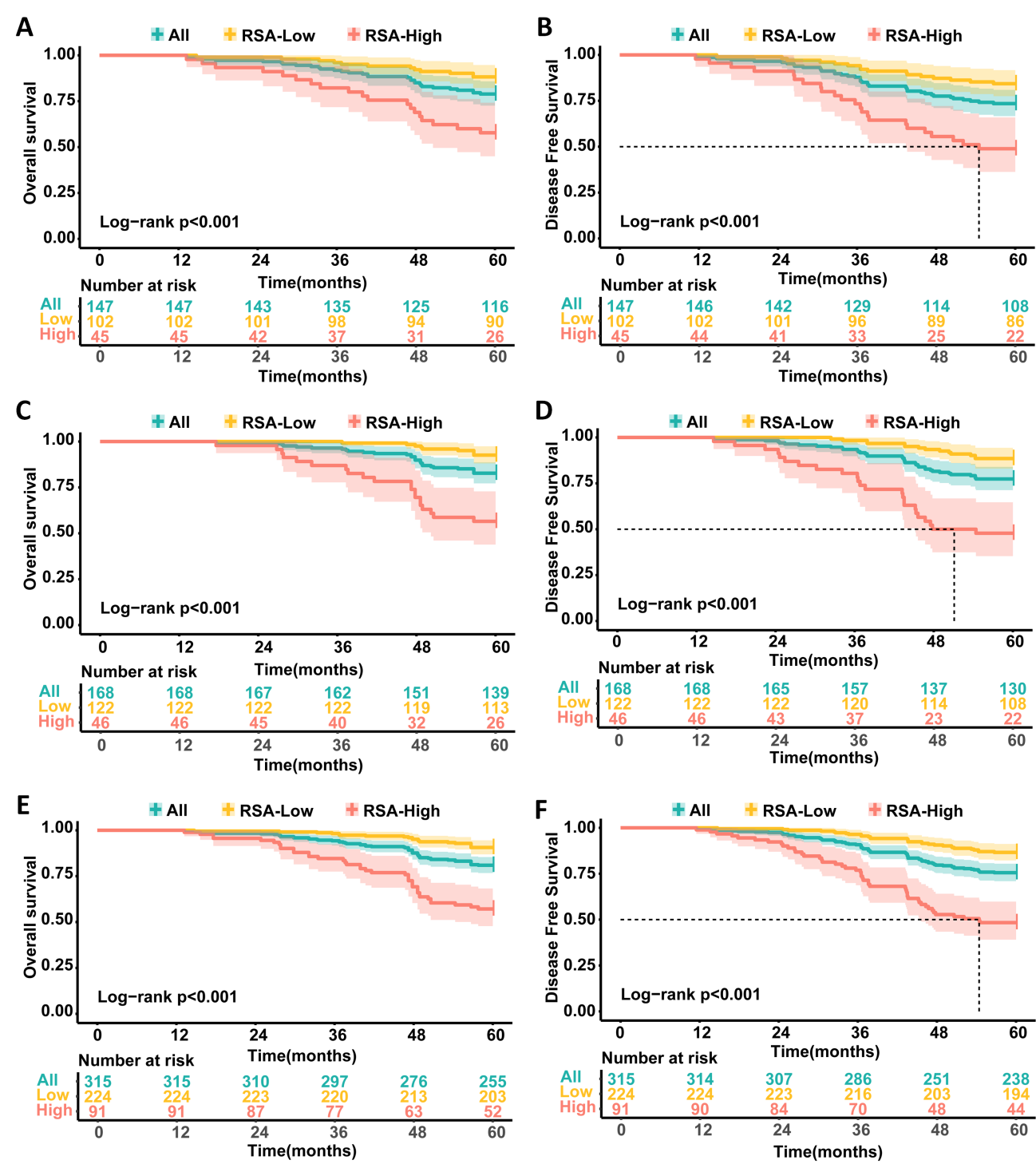


**Supplementary Figure 11**. **Survival curves based on low- and high-risk stratification using the eCura system and RSA model across different datasets of peripheral blood samples. A. 5-year OS curve for the peripheral blood training cohort; B. 5-year DFS curve for the peripheral blood training cohort; C. 5-year OS curve for the peripheral blood validation cohort; D. 5-year DFS curve for the peripheral blood validation cohort; E. 5-year OS curve for the combined dataset (training + validation cohorts) of peripheral blood samples; F. 5-year DFS curve for the combined dataset (training + validation cohorts) of peripheral blood samples.**


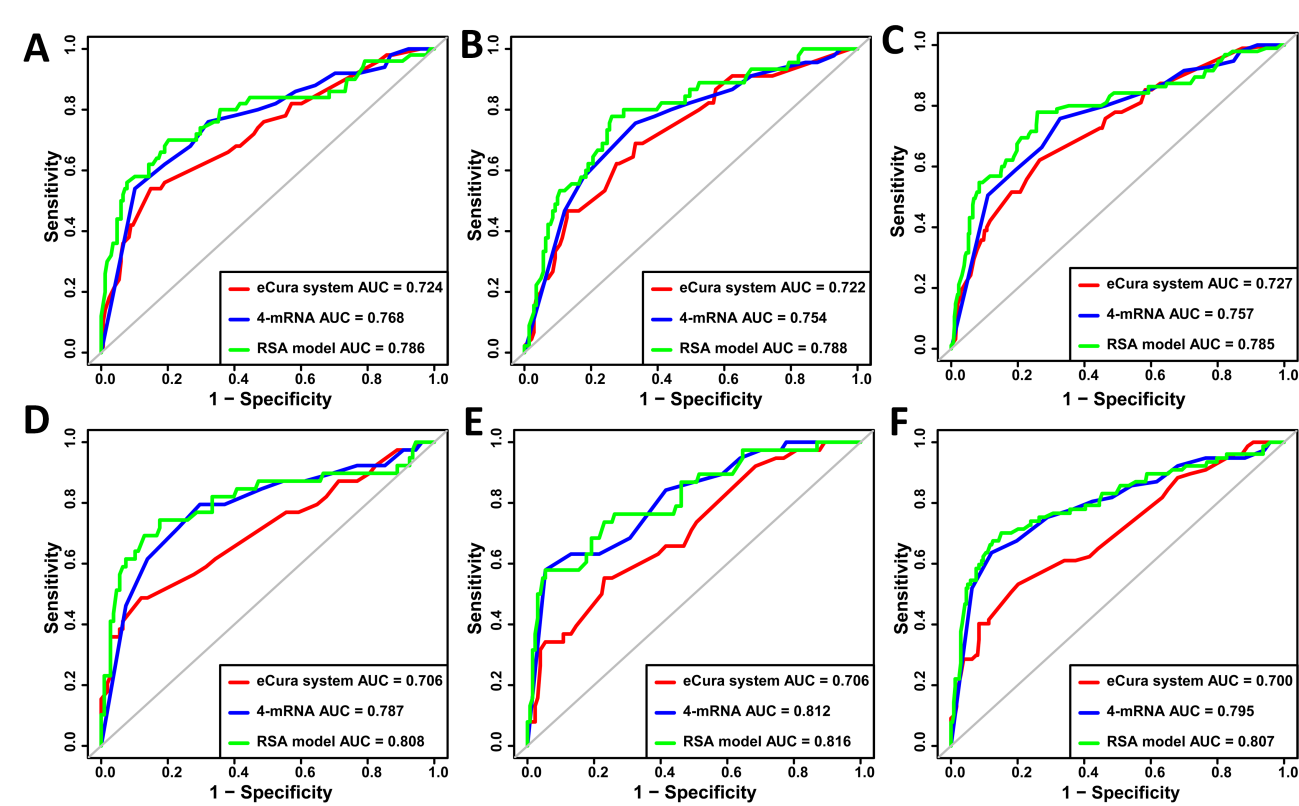


**Supplementary Figure 12. ROC curves for predicting DFS in T1 gastric cancer patients using different models. A. Tissue sample training cohort; B. Tissue sample validation cohort; C. Combined tissue sample dataset; D. Peripheral blood sample training cohort; E. Peripheral blood sample validation cohort; F. Combined peripheral blood sample dataset.**

**
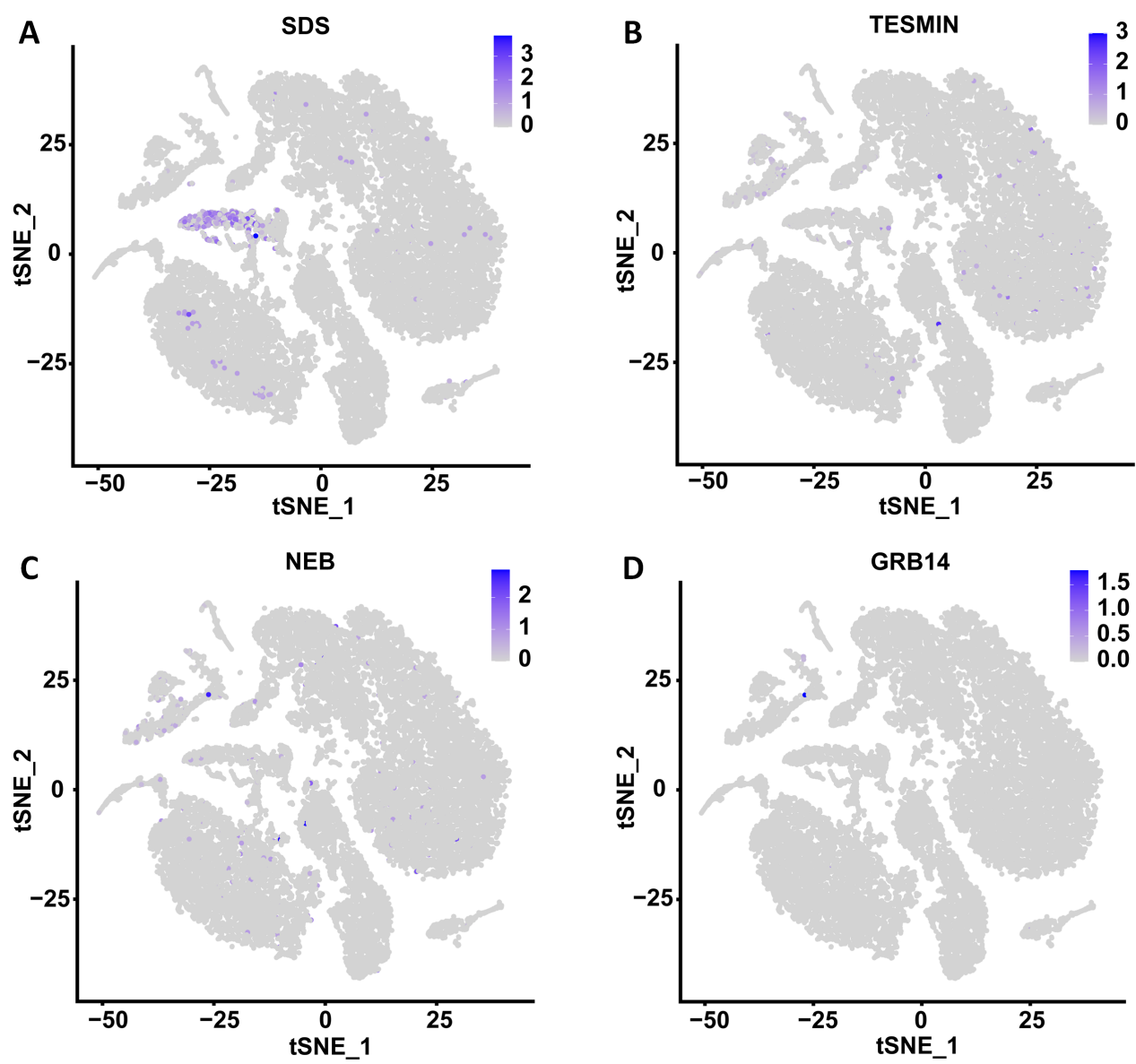
**

**Supplementary Figure 13. Expression distribution of four candidate genes in the tumor microenvironment. (A) SDS; (B) TESMIN; (C) NEB; (D) GRB14.**

**Supplementary Tables**

**Supplementary Table 1 Clinical characteristics of cohorts examining candidate mRNA expression in surgical resection specimens and peripheral blood specimens**

| **Clinical characteristic** | **Surgical specimen cohort (N=28)** | **Peripheral blood specimen cohort (N=22)** |
| --- | --- | --- |
| **Gender** |  |  |
| Male | 17 (60.7%) | 14 (63.6%) |
| Female | 11 (39.3%) | 8 (36.4%) |
| **Age(years)** |  |  |
| ≤65 | 19 (67.9%) | 12 (54.5%) |
| ＞65 | 9 (32.1%) | 10 (45.5%) |
| **BMI(Kg/m^2^)** |  |  |
| ≤25 | 21(75.0%) | 15 (68.2%) |
| ＞25 | 7(25.0%) | 7 (31.8%) |
| **Primary site** |  |  |
| Up 1/3 | 10 (35.7%) | 8 (36.4%) |
| Middle 1/3 | 6 (21.4%) | 4 (18.2%) |
| Lower 1/3 | 12 (42.9%) | 10 (45.4%) |
| **Tumor size(cm)** |  |  |
| ≤3 | 11 (39.3%) | 9 (40.9%) |
| ＞3 | 17 (60.7%) | 13 (59.1%) |
| **Histology** |  |  |
| None/Low | 16 (57.1%) | 13 (59.1%) |
| High/Median | 12 (42.9%) | 9 (40.9%) |
| **Infiltration depth** |  |  |
| M/SM1 | 7 (25.0%) | 4 (18.2%) |
| SM2 | 21 (75.0%) | 18 (81.8%) |
| **Vascular invasion** |  |  |
| Yes | 13 (46.4%) | 6 (27.3%) |
| No | 15 (53.6%) | 16 (72.7%) |
| **Lymphatic invasion** |  |  |
| Yes | 11 (39.3%) | 10 (45.5%) |
| No | 17 (60.7%) | 12 (54.5%) |

**Supplementary Table 2 Clinical characteristics of training set and validation set of surgical resection specimen cohort[n(%)]**

| **Clinical characteristic** | **Training cohort**  **(N=218)** | **Validation cohort**  **(N=186)** | **P value** |
| --- | --- | --- | --- |
| **Gender** |  |  | 0.780 |
| Male | 133 (61.0%) | 116 (62.4%) |  |
| Female | 85 (39.0%) | 70 (37.6%) |  |
| **Age(years)** |  |  | 0.732 |
| ≤65 | 173 (79.4%) | 145 (78.0%) |  |
| ＞65 | 45 (20.6%) | 41 (22.0%) |  |
| **BMI(Kg/m^2^)** |  |  | 0.955 |
| ≤25 | 151 (69.3%) | 127 (68.3%) |  |
| ＞25 | 67 (30.7%) | 59 (31.7%) |  |
| **Primary site** |  |  | 0.421 |
| Up 1/3 | 71 (32.6%) | 60 (32.3%) |  |
| Middle 1/3 | 48 (22.0%) | 32 (17.2%) |  |
| Lower 1/3 | 99 (45.4%) | 94 (50.5%) |  |
| **Tumor size(cm)** |  |  | 0.683 |
| ≤3 | 97 (44.5%) | 79 (42.5%) |  |
| ＞3 | 121(55.5%) | 107 (57.5%) |  |
| **Histology** |  |  | 0.672 |
| None/Low | 194 (89.0%) | 163 (87.6%) |  |
| High/Median | 24 (11.0%) | 23 (12.4%) |  |
| **Infiltration depth** |  |  | 0.977 |
| M/SM1 | 138 (63.3%) | 118 (63.4%) |  |
| SM2 | 80 (36.7%) | 68 (36.6%) |  |
| **Vascular invasion** |  |  | 0.883 |
| Yes | 70 (32.1%) | 61 (32.8%) |  |
| No | 148 (67.9%) | 125 (67.2%) |  |
| **Lymphatic invasion** |  |  | 0.519 |
| Yes | 65 (29.8%) | 61 (32.8%) |  |
| No | 153 (70.2%) | 125 (67.2%) |  |
| **N stage** |  |  | 0.770 |
| N0 | 184 (84.4%) | 155 (83.3%) |  |
| N+ | 34 (15.6%) | 31 (16.7%) |  |

**Supplementary Table 3 Clinical characteristics of the endoscopic biopsy specimen validation set cohort[n(%)]**

| **Clinical characteristic** | **Training cohort**  **(N=122)** |
| --- | --- |
| **Gender** |  |
| Male | 79 (64.8%) |
| Female | 43 (35.2%) |
| **Age(years)** |  |
| ≤65 | 95 (77.9%) |
| ＞65 | 27 (22.1%) |
| **BMI(Kg/m^2^)** |  |
| ≤25 | 75 (61.5%) |
| ＞25 | 47 (38.5%) |
| **Primary site** |  |
| Up 1/3 | 41 (33.6%) |
| Middle 1/3 | 24 (19.7%) |
| Lower 1/3 | 57 (46.7%) |
| **Tumor size(cm)** |  |
| ≤3 | 52 (42.6%) |
| ＞3 | 70 (57.4%) |
| **Histology** |  |
| None/Low | 108 (88.5%) |
| High/Median | 14 (11.5%) |
| **Infiltration depth** |  |
| M/SM1 | 78 (63.9%) |
| SM2 | 44 (36.1%) |
| **Vascular invasion** |  |
| Yes | 39 (32.0%) |
| No | 83 (68.0%) |
| **Lymphatic invasion** |  |
| Yes | 42 (34.4%) |
| No | 80 (65.6%) |
| **N stage** |  |
| N0 | 104 (85.2%) |
| N+ | 18 (14.8%) |

**Supplementary Table 4 Clinical characteristics of peripheral blood specimen cohorts in training set and validation set[n(%)]**

| **Clinical characteristic** | **Training cohort**  **(N=147)** | **Validation cohort**  **(N=168)** | **Prospective cohort**  **(N=97)** | **P value** |
| --- | --- | --- | --- | --- |
| **Gender** |  |  |  | 0.941 |
| Male | 93 (63.3%) | 104 (61.9%) | 62 (63.9%) |  |
| Female | 54 (36.7%) | 64 (38.1%) | 35 (36.1%) |  |
| **Age(years)** |  |  |  | 0.834 |
| ≤65 | 112 (76.2%) | 129(76.8%) | 77 (79.4%) |  |
| ＞65 | 35 (23.8%) | 39(23.2%) | 20 (20.6%) |  |
| **BMI(Kg/m^2^)** |  |  |  | 0.576 |
| ≤25 | 93 (63.3%) | 100 (59.5%) | 55 (56.7%) |  |
| ＞25 | 54 (36.7%) | 68 (40.5%) | 42 (43.3%) |  |
| **Primary site** |  |  |  | 0.999 |
| Up 1/3 | 55 (37.4%) | 63 (37.5%) | 37 (38.1%) |  |
| Middle 1/3 | 32 (21.8%) | 38 (22.6%) | 22 (22.7%) |  |
| Lower 1/3 | 60 (40.8%) | 67 (39.9%) | 38 (39.2%) |  |
| **Tumor size(cm)** |  |  |  | 0.948 |
| ≤3 | 63 (42.9%) | 71(42.3%) | 40 (41.2%) |  |
| ＞3 | 84 (57.1%) | 97(57.7%) | 57 (58.8%) |  |
| **Histology** |  |  |  | 0.912 |
| None/Low | 126 (85.7%) | 145 (86.3%) | 85 (87.6%) |  |
| High/Median | 21 (14.3%) | 23 (13.7%) | 12(12.4%) |  |
| **Infiltration depth** |  |  |  | 0.994 |
| M/SM1 | 93 (63.3%) | 107 (63.7%) | 62 (63.9%) |  |
| SM2 | 54 (36.7%) | 61 (36.3%) | 35 (36.1%) |  |
| **Vascular invasion** |  |  |  | 0.697 |
| Yes | 48 (32.7%) | 54 (32.1%) | 27 (27.8%) |  |
| No | 99 (67.3%) | 114 (67.9%) | 70 (72.2%) |  |
| **Lymphatic invasion** |  |  |  | 0.922 |
| Yes | 50 (34.0%) | 54 (32.1%) | 31 (32.0%) |  |
| No | 97 (66.0%) | 114 (67.9%) | 66 (68.0%) |  |
| **N stage** |  |  |  | 0.964 |
| N0 | 125 (85.0%) | 141 (83.9%) | 82 (84.5%) |  |
| N+ | 22 (15.0%) | 27 (16.1%) | 15 (15.5%) |  |

**Supplementary Table 5. The primers for four panel genes**

| Name | Sequence |
| --- | --- |
| TESMIN_F | TCCCCTCGGGATCAACTCTT |
| TESMIN_R | AGGTGTGCAGTGAAGCTCAT |
| NEB_F | GGATGTCTCACCAGGAACGG |
| NEB_R | GTCCCCTTGCTCAAGTTCTC |
| GRB14_F | GTCCATGACCTACGAGACAGG |
| GRB14_R | GAGCCGTTATGTCACTGGGT |
| SDS_F | TCAGACCCATCACCTTTGCC |
| SDS_R | AATGTGCACAGCCTTGCTTG |
| F:Forward | R:Reverse |
| The conditions for RNA extraction | Take part of the tissue and add 1ml TRIzol reagent, place it on ice and grind thoroughly. Subsequently adding 1/5 volume of chloroform to the mixture, mix by inverting upside down, and place the sample on ice for 5 minutes. Centrifige at 12.000 rpm at high speed, 4℃ for 15 minutes. After l5 minutes, absorb the supernatant and add an equal volume of isopropyl alcohol. Mix by inverting and let stand on ice for 10 minutes. Centrifuge again at high speed, 12000 rpm, 4℃, for 10 minutes. After discarding the supernatant, add 400 ul of 75% ethanol, centrifuge at 9000 rpm, 4℃ for 5 minutes, Discard the supernatant and after the precipitate is dried, add an appropriate amount of DEPC water to dissolve the precipitate. |
|  |  |
| The PCR conditions | First set the pre-denaturation program to 95℃, 10 minutes. Afterwards, 40 cycles of reaction were carried out, with each cycle ending at 95℃ for 15 seconds for denaturation, 58℃ for 30 seconds for annealing, and 72℃ for 30 seconds for extension. Finally, set the dissolution curve program to 95℃, terminate the extension in 15 seconds, maintain the system temperature at 60℃ for 1 min. And finally increase the temperature by 0.3℃ per unit time to 95℃ for 15s. |

**Supplementary Table 6. Relationship between the expression of four candidate mRNAs and clinical characteristics
in the pilot cohort of tissue specimens**

| **Clinical characteristic** | **SDS** | | **P value** | **TESMIN** | | **P value** | **NEB** | | **P value** | **GRB14** | | **P value** |
| --- | --- | --- | --- | --- | --- | --- | --- | --- | --- | --- | --- | --- |
|  | **High** | **Low** |  | **High** | **Low** |  | **High** | **Low** |  | **High** | **Low** |  |
| **Gender** |  |  | 1.000* |  |  | 0.934 |  |  | 0.248* |  |  | 0.701* |
| Male | 10 (58.8%) | 7 (63.6%) |  | 9 (60.0%) | 8 (61.5%) |  | 12 (70.6%) | 5 (45.5%) |  | 11 (64.7%) | 6 (54.5%) |  |
| Female | 7 (41.2%) | 4 (36.4%) |  | 6 (40.0%) | 5 (38.5%) |  | 5 (29.4%) | 6 (54.5%) |  | 6 (34.3%) | 5 (45.5%) |  |
| **Age(years)** |  |  | 1.000* |  |  | 1.000* |  |  | 0.249* |  |  | 0.249* |
| ≤65 | 12 (70.6%) | 7 (63.6%) |  | 10 (66.7%) | 9 (69.2%) |  | 10 (58.8%) | 9 (81.8%) |  | 10 (58.8%) | 9 (81.8%) |  |
| ＞65 | 5 (29.4%) | 4 (36.4%) |  | 5 (33.3%) | 4 (30.8%) |  | 7 (41.2%) | 2 (18.2%) |  | 7 (41.2%) | 2 (18.2%) |  |
| **Tumor size(cm)** |  |  | 1.000* |  |  | 0.003 |  |  | 0.248* |  |  | 0.115* |
| ≤3 | 7 (41.2%) | 4 (36.4%) |  | 2 (13.3%) | 9 (69.2%) |  | 5 (29.4%) | 6 (54.5%) |  | 9 (52.9%) | 2 (18.2%) |  |
| ＞3 | 10 (58.8%) | 7 (63.6%) |  | 13 (86.7%) | 4 (30.8%) |  | 12 (70.6%) | 5 (45.5%) |  | 8 (47.1%) | 9 (81.8%) |  |
| **Histology** |  |  | 0.441* |  |  | 0.001 |  |  | 1.000* |  |  | 0.121* |
| None/Low | 11 (64.7%) | 5 (45.5%) |  | 13 (86.7%) | 3 (23.1%) |  | 10 (58.8%) | 6 (54.5%) |  | 12 (70.6%) | 4 (36.4%) |  |
| High/Median | 6 (35.3%) | 6 (54.5%) |  | 2 (13.3%) | 10 (76.9%) |  | 7 (41.2%) | 5 (45.5%) |  | 5 (29.4%) | 7 (63.6%) |  |
| **Infiltration depth** |  |  | 0.683* |  |  | 0.198* |  |  | 0.668* |  |  | 0.076* |
| M/SM1 | 4 (23.5%) | 3 (27.3%) |  | 2 (13.3%) | 5 (38.5%) |  | 5 (29.4%) | 2 (18.2%) |  | 2 (11.8%) | 5 (45.5%) |  |
| SM2 | 13 (76.5%) | 8 (72.7%) |  | 13 (86.7%) | 8 (61.5%) |  | 12 (70.6%) | 9 (81.8%) |  | 15 (88.2%) | 6 (54.5%) |  |
| **Vascular invasion** |  |  | 0.390 |  |  | 0.431 |  |  | 0.390 |  |  | 0.390 |
| Yes | 9 (53.0%) | 4 (36.4%) |  | 8 (53.3%) | 5 (38.5%) |  | 9 (52.9%) | 4 (36.4%) |  | 9 (52.9%) | 4 (36.4%) |  |
| No | 8 (47.0%) | 7 (63.6%) |  | 7 (46.7%) | 8 (61.5%) |  | 8 (47.1%) | 7 (63.6%) |  | 8 (47.1%) | 7 (63.6%) |  |
| **Lymphatic invasion** |  |  | 0.115* |  |  | 0.390 |  |  | 0.435 |  |  | 0.435 |
| Yes | 9 (53.0%) | 2 (18.2%) |  | 7 (46.7%) | 4 (30.8%) |  | 8 (47.1%) | 3 (27.3%) |  | 8 (47.1%) | 3 (27.3%) |  |
| No | 8 (47.0%) | 9 (81.8%) |  | 8 (53.3%) | 9 (69.2%) |  | 9 (52.9%) | 8 (72.7%) |  | 9 (52.9%) | 8 (72.7%) |  |

**Note: *Fisher test.**

**Supplementary Table 7. Relationship between the expression of four candidate mRNAs and clinical characteristics
in the pilot cohort of peripheral blood specimens**

| **Clinical characteristic** | **SDS** | | **P value** | **TESMIN** | | **P value** | **NEB** | | **P value** | **GRB14** | | **P value** |
| --- | --- | --- | --- | --- | --- | --- | --- | --- | --- | --- | --- | --- |
|  | **High** | **Low** |  | **High** | **Low** |  | **High** | **Low** |  | **High** | **Low** |  |
| **Gender** |  |  | 1.000* |  |  | 1.000* |  |  | 1.000* |  |  | 1.000* |
| Male | 9 (64.3%) | 5 (62.5%) |  | 10 (66.7%) | 4 (57.1%) |  | 8 (66.7%) | 6 (60.0%) |  | 7 (63.6%) | 7 (63.6%) |  |
| Female | 5 (35.7%) | 3 (37.5%) |  | 5 (33.3%) | 3 (42.9%) |  | 4 (33.3%) | 4 (40.0%) |  | 4 (36.4%) | 4 (36.4%) |  |
| **Age(years)** |  |  | 1.000* |  |  | 0.652* |  |  | 1.000* |  |  | 0.670 |
| ≤65 | 8 (57.1%) | 4 (50.0%) |  | 9 (60.0%) | 3 (42.9%) |  | 7 (58.3%) | 5 (50.0%) |  | 5 (45.5%) | 6 (54.5%) |  |
| ＞65 | 6 (42.9%) | 4 (50.0%) |  | 6 (40.0%) | 4 (57.1%) |  | 5 (41.7%) | 5 (50.0%) |  | 6 (54.5%) | 5 (45.5%) |  |
| **Tumor size(cm)** |  |  | 0.001* |  |  | 0.007* |  |  | 0.027* |  |  | 0.080* |
| ≤3 | 2 (14.3%) | 7 (87.5%) |  | 3 (20.0%) | 6 (85.7%) |  | 2 (16.7%) | 7 (70.0%) |  | 2 (18.2%) | 7 (63.6%) |  |
| ＞3 | 12 (85.7%) | 1 (12.5%) |  | 12 (80.0%) | 1 (14.3%) |  | 10 (83.3%) | 3 (30.0%) |  | 9 (81.8%) | 4 (36.4%) |  |
| **Histology** |  |  | 0.662* |  |  | 0.376* |  |  | 0.222* |  |  | 0.080* |
| None/Low | 9 (64.3%) | 4 (50.0%) |  | 10 (66.7%) | 3 (42.9%) |  | 9 (75.0%) | 4 (40.0%) |  | 9 (81.8%) | 4 (36.4%) |  |
| High/Median | 5 (35.7%) | 4 (50.0%) |  | 5 (33.3%) | 4 (57.1%) |  | 3 (25.0%) | 6 (60.0%) |  | 2 (18.2%) | 7 (63.6%) |  |
| **Infiltration depth** |  |  | 0.602* |  |  | 0.077* |  |  | 0.293 |  |  | 0.586* |
| M/SM1 | 2 (14.3%) | 2 (25.0%) |  | 1 (6.7%) | 3 (42.9%) |  | 1 (8.3%) | 3 (30.0%) |  | 1 (9.1%) | 3 (27.3%) |  |
| SM2 | 12 (85.7%) | 6 (75.0%) |  | 14 (93.3%) | 4 (57.1%) |  | 11 (91.7%) | 7 (70.0%) |  | 10 (90.9%) | 8 (72.7%) |  |
| **Vascular invasion** |  |  | 1.000* |  |  | 0.616* |  |  | 0.162* |  |  | 0.149* |
| Yes | 4 (28.6%) | 2 (25.0%) |  | 5 (33.3%) | 1 (14.3%) |  | 5 (41.7%) | 1 (10.0%) |  | 5 (45.5%) | 1 (9.1%) |  |
| No | 10 (71.4%) | 6 (75.0%) |  | 10 (66.7%) | 6 (85.7%) |  | 7 (58.3%) | 9 (90.0%) |  | 6 (54.5%) | 10 (90.9%) |  |
| **Lymphatic invasion** |  |  | 0.204* |  |  | 1.000* |  |  | 0.231* |  |  | 0.010 |
| Yes | 8 (57.1%) | 2 (25.0%) |  | 7 (46.7%) | 3 (42.9%) |  | 7 (58.3%) | 3 (30.0%) |  | 8 (72.7%) | 2 (18.2%) |  |
| No | 6 (42.9%) | 6 (75.0%) |  | 8 (53.3%) | 4 (57.1%) |  | 5 (41.7%) | 7 (70.0%) |  | 3 (27.3%) | 9 (81.8%) |  |

**Note: *Fisher test.**

**Supplementary Table 8. Multifactorial logistic regression analysis of 4 candidate mRNAs influencing LNM in T1 GC patients**

| **Surgical resection specimens--training set** | | | |
| --- | --- | --- | --- |
| mRNA | OR | 95%CI | P value |
| SDS (High vs. Low) | 3.151 | 1.066-9.313 | 0.038 |
| TESMIN (High vs. Low) | 7.531 | 2.933-33.110 | 0.003 |
| NEB (High vs. Low) | 5.970 | 1.651-21.589 | 0.006 |
| GRB14 (High vs. Low) | 5.853 | 1.862-18.401 | 0.003 |
| **Peripheral blood specimens--training set** | | | |
| mRNA | OR | 95%CI | P value |
| SDS (High vs. Low) | 6.176 | 1.250-30.523 | 0.026 |
| TESMIN (High vs. Low) | 14.005 | 1.708-114.819 | 0.014 |
| NEB (High vs. Low) | 7.871 | 1.604-38.613 | 0.011 |
| GRB14 (High vs. Low) | 8.904 | 1.834-42.227 | 0.007 |

**Supplementary Table 9. Multifactorial logistic regression analysis affecting LNM in T1 GC patients**

| **Surgical resection specimens--training set** | | | |
| --- | --- | --- | --- |
| Variables | OR | 95%CI | P value |
| Infiltration depth (SM2 vs. M/SM1) | 5.940 | 1.814-19.452 | 0.003 |
| Tumour size (≥3cm vs. ＜3cm) | 5.906 | 1.673-20.856 | 0.006 |
| Lymphatic invasion (Yes vs. No) | 5.935 | 1.767-19.935 | 0.004 |
| 4-mRNA panel (High vs. Low) | 13.911 | 4.585-42.212 | ＜0.001 |
| **Peripheral blood specimens--training set** | | | |
| Variables | OR | 95%CI | P value |
| Infiltration depth (SM2 vs. M/SM1) | 5.632 | 1.652-15.677 | 0.016 |
| Tumour size (≥3cm vs. ＜3cm) | 4.608 | 1.234-11.539 | 0.023 |
| Lymphatic invasion (Yes vs. No) | 3.794 | 1.124-9.689 | 0.020 |
| 4-mRNA panel (High vs. Low) | 9.894 | 2.244-45.523 | ＜0.001 |

**Supplementary Table 10. Comparison of performance indicators of different models for predicting LNM in tissue specimens of T1 GC patients**

| **Variable** | **AUC** | **Accuracy** | **Sensitivity** | **Specificity** | **PPV** | **NPV** | **PLR (x10)** | **NLR** | **F1 score** |
| --- | --- | --- | --- | --- | --- | --- | --- | --- | --- |
| **Training set** |  |  |  |  |  |  |  |  |  |
| Clinical features | 0.820 | 0.766 | 0.735 | 0.772 | 0.373 | 0.940 | 0.322 | 0.343 | 0.495 |
| 4-mRNA panel | 0.838 | 0.761 | 0.823 | 0.750 | 0.378 | 0.958 | 0.329 | 0.235 | 0.519 |
| RSA model | 0.890 | 0.871 | 0.765 | 0.891 | 0.565 | 0.953 | 0.703 | 0.264 | 0.65 |
| **Validation set** |  |  |  |  |  |  |  |  |  |
| Clinical features | 0.796 | 0.742 | 0.774 | 0.735 | 0.369 | 0.942 | 0.293 | 0.307 | 0.500 |
| 4-mRNA panel | 0.835 | 0.806 | 0.774 | 0.812 | 0.453 | 0.947 | 0.414 | 0.278 | 0.571 |
| RSA model | 0.878 | 0.839 | 0.839 | 0.839 | 0.510 | 0.963 | 0.520 | 0.192 | 0.634 |

Note: AUC, area under the curve; PPV, Positive Predictive Value; NVP, Negative Predictive Value; PLR, Positive Likelihood Ratio; NLR, Negative Likelihood Ratio.

**Supplementary Table 11. Comparison of performance indicators of different models for predicting LNM in endoscopic biopsy specimens of T1 GC patients**

| **Variable** | **AUC** | **Accuracy** | **Sensitivity** | **Specificity** | **PPV** | **NPV** | **PLR (x10)** | **NLR** | **F1 score** |
| --- | --- | --- | --- | --- | --- | --- | --- | --- | --- |
| Clinical features | 0.829 | 0.787 | 0.722 | 0.798 | 0.382 | 0.943 | 0.358 | 0.348 | 0.500 |
| 4-mRNA panel | 0.890 | 0.730 | 0.944 | 0.692 | 0.347 | 0.986 | 0.307 | 0.080 | 0.507 |
| RSA model | 0.928 | 0.844 | 0.833 | 0.846 | 0.484 | 0.967 | 0.542 | 0.197 | 0.612 |

Note: AUC, area under the curve; PPV, Positive Predictive Value; NVP, Negative Predictive Value; PLR, Positive Likelihood Ratio; NLR, Negative Likelihood Ratio.

**Supplementary Table 12. Comparison of performance indicators of different models for predicting LNM in patients with T1 GC in peripheral blood samples**

| **Variable** | **AUC** | **Accuracy** | **Sensitivity** | **Specificity** | **PPV** | **NPV** | **PLR (x10)** | **NLR** | **F1 score** |
| --- | --- | --- | --- | --- | --- | --- | --- | --- | --- |
| **Training set** |  |  |  |  |  |  |  |  |  |
| Clinical features | 0.799 | 0.762 | 0.636 | 0.784 | 0.341 | 0.925 | 0.295 | 0.464 | 0.444 |
| 4-mRNA panel | 0.844 | 0.694 | 0.909 | 0.656 | 0.317 | 0.976 | 0.264 | 0.139 | 0.471 |
| RSA model | 0.873 | 0.857 | 0.818 | 0.864 | 0.514 | 0.964 | 0.602 | 0.210 | 0.632 |
| **Validation set** |  |  |  |  |  |  |  |  |  |
| Clinical features | 0.760 | 0.690 | 0.815 | 0.667 | 0.319 | 0.949 | 0.244 | 0.278 | 0.458 |
| 4-mRNA panel | 0.820 | 0.732 | 0.815 | 0.716 | 0.355 | 0.953 | 0.287 | 0.259 | 0.494 |
| RSA model | 0.852 | 0.798 | 0.815 | 0.794 | 0.431 | 0.957 | 0.396 | 0.233 | 0.564 |

Note: AUC, area under the curve; PPV, Positive Predictive Value; NVP, Negative Predictive Value; PLR, Positive Likelihood Ratio; NLR, Negative Likelihood Ratio.

**Supplementary Table 13. Comparison of performance indicators of different models for predicting LNM in T1 GC patients in the prospective cohort**

| **Variable** | **AUC** | **Accuracy** | **Sensitivity** | **Specificity** | **PPV** | **NPV** | **PLR (x10)** | **NLR** | **F1 score** |
| --- | --- | --- | --- | --- | --- | --- | --- | --- | --- |
| Clinical features | 0.707 | 0.66 | 0.667 | 0.659 | 0.263 | 0.915 | 0.195 | 0.506 | 0.377 |
| 4-mRNA panel | 0.788 | 0.515 | 1.000 | 0.427 | 0.243 | 1.000 | 0.174 | 0.000 | 0.390 |
| RSA model | 0.812 | 0.763 | 0.800 | 0.756 | 0.375 | 0.954 | 0.328 | 0.265 | 0.511 |

Note: AUC, area under the curve; PPV, Positive Predictive Value; NVP, Negative Predictive Value; PLR, Positive Likelihood Ratio; NLR, Negative Likelihood Ratio.
